# The potential impact of food taxes and subsidies on cardiovascular disease and diabetes burden and disparities in the United States

**DOI:** 10.1186/s12916-017-0971-9

**Published:** 2017-11-27

**Authors:** José L. Peñalvo, Frederick Cudhea, Renata Micha, Colin D. Rehm, Ashkan Afshin, Laurie Whitsel, Parke Wilde, Tom Gaziano, Jonathan Pearson-Stuttard, Martin O’Flaherty, Simon Capewell, Dariush Mozaffarian

**Affiliations:** 1 Friedman School of Nutrition Science & Policy, Tufts University, 150 Harrison Ave, Boston, MA 02111 USA; 2 Montefiore Medical Center, New York, NY 10467 USA; 3 Institute for Health Metrics and Evaluation (IHME) at the University of Washington, Seattle, WA 98121 USA; 4 American Heart Association (AHA), Washington, DC 20036 USA; 5 Divisions of Cardiovascular Medicine, Brigham and Women’s Hospital, Boston, MA 02115 USA; 6 School of Public Health, Imperial College London, London, W2 1PG UK; 7 Department of Public Health and Policy, University of Liverpool, Liverpool, L69 3GL UK

**Keywords:** Cardiovascular disease, Diabetes, Diet, Taxes, Subsidies, Policy, Cardiometabolic, Disparities

## Abstract

**Background:**

Fiscal interventions are promising strategies to improve diets, reduce cardiovascular disease and diabetes (cardiometabolic diseases; CMD), and address health disparities. The aim of this study is to estimate the impact of specific dietary taxes and subsidies on CMD deaths and disparities in the US.

**Methods:**

Using nationally representative data, we used a comparative risk assessment to model the potential effects on total CMD deaths and disparities of price subsidies (10%, 30%) on fruits, vegetables, whole grains, and nuts/seeds and taxes (10%, 30%) on processed meat, unprocessed red meats, and sugar-sweetened beverages. We modeled two gradients of price-responsiveness by education, an indicator of socioeconomic status (SES), based on global price elasticities (18% greater price-responsiveness in low vs. high SES) and recent national experiences with taxes on sugar-sweetened beverages (65% greater price-responsiveness in low vs. high SES).

**Results:**

Each price intervention would reduce CMD deaths. Overall, the largest proportional reductions were seen in stroke, followed by diabetes and coronary heart disease. Jointly altering prices of all seven dietary factors (10% each, with 18% greater price-responsiveness by SES) would prevent 23,174 (95% UI 22,024–24,595) CMD deaths/year, corresponding to 3.1% (95% UI 2.9–3.4) of CMD deaths among Americans with a lower than high school education, 3.6% (95% UI 3.3–3.8) among high school graduates/some college, and 2.9% (95% UI 2.7–3.5) among college graduates. Applying a 30% price change and larger price-responsiveness (65%) in low SES, the corresponding reductions were 10.9% (95% UI 9.2–10.8), 9.8% (95% UI 9.1–10.4), and 6.7% (95% UI 6.2–7.6). The latter scenario would reduce disparities in CMD between Americans with lower than high school versus a college education by 3.5 (95% UI 2.3–4.5) percentage points.

**Conclusions:**

Modest taxes and subsidies for key dietary factors could meaningfully reduce CMD and improve US disparities.

**Electronic supplementary material:**

The online version of this article (doi:10.1186/s12916-017-0971-9) contains supplementary material, which is available to authorized users.

## Background

Cardiovascular disease (CVD) remains the leading cause of disability and death in the US and globally [1], with associated economic costs projected to increase substantially along with population aging [2]. Additionally, the risk of type 2 diabetes is steadily increasing, with tremendous associated health and economic consequences [3]. Large disparities in these burdens are also evident, with a much higher risk among those of lower socioeconomic status (SES) [4, 5] and disparities growing over time [6]. Based on health burdens, economic costs, and corresponding inequities, the identification of effective population interventions to reduce cardiovascular disease and diabetes (cardiometabolic diseases, CMD), as well as disparities, is crucial.

A suboptimal diet is a major cause of CMD [7]. Among strategies to improve dietary behaviors, fiscal interventions to alter food prices are promising [8]. Such approaches may not only improve population health, but also potentially reduce disparities in diet quality and diet-related health burdens [9]. Several fiscal strategies, such as taxation of sugar-sweetened beverages (SSB) and/or unhealthy snacks [10–12] and subsidization of fruits and vegetables [13], have been already implemented. Yet, while such measures significantly improve diet, neither the potential impact of such interventions on CMD in the US, nor the potential impact on disparities, has been quantified. In addition, other foods beyond fruits and vegetables or SSBs represent appealing targets for fiscal interventions, but the separate and joint benefits of such approaches have not been assessed. To address these knowledge gaps and inform policy-makers, we quantified the impact of altering the intakes of seven key food groups through economic incentives on coronary heart disease (CHD), stroke, and type 2-diabetes mortality in the US, as well as the impact on corresponding disparities.

## Methods

### Study design

We utilized nationally representative US data in a comparative risk assessment framework. We incorporated national data from 2012 on the consumption of selected food items, by age, sex, and SES; estimates of etiological effects of these foods on CMD, by age; observed national CMD deaths, by age, sex, and SES; and estimated impact of pricing changes on dietary habits, by SES. Because data on income is not routinely collected in US mortality datasets, we used educational attainment as a measure of SES. Using the National Health and Nutrition Examination Survey (NHANES) [14], the income-to-poverty ratio was mapped against educational levels (lower than high school (< HS), high school or some college (HS), college graduate (COL)), confirming that education is a reasonable proxy measure (Additional file 1: Figure S1).

### Distributions of dietary targets

Current dietary intakes were obtained from NHANES [14], combining the two cycles (2009–2010, 2011–2012; N = 8516 individuals) to increase statistical precision among population subgroups. We used survey-weights to obtain representative data for non-institutionalized US adults (age 25+) based on the average of two non-consecutive 24-hour dietary recalls, accounting for within-person variation and adjusting for total energy intake using the residual method [15] to reduce measurement error and account for individual variability. Current distributions of body mass index (BMI) were also obtained from NHANES (2009–2012). Data were obtained as mean and SD for each dietary target and BMI, stratified by age groups (25–34, 35–44, 45–54, 55–64, 65–74, 75+ years), sex, and education (< HS, HS, and COL) (Additional file 1: Table S1).

### Etiologic effects of dietary changes

We defined seven foods [16] based on the evidence of their association with cardiometabolic outcomes [4] and policy interest [17]; these were fruits, vegetables, whole grains, nuts/seeds, SSBs, and processed and unprocessed red meat (Table 1). We focused on price interventions on foods rather than isolated nutrients (e.g. sodium, added sugars) given the evidence on the importance of overall dietary patterns in health [4, 17], and also considering the practical challenges of taxing/subsidizing isolated nutrients. For each food, the evidence for, and magnitude and uncertainty of the etiologic effect was compiled from meta-analyses of prospective cohorts or randomized trials [18] using previously developed methods [19]. All etiologic effects incorporated declining proportional effects (i.e., relative risks, RRs) by age [18, 20].

**Table 1:** Selected dietary factors, price-responsiveness in consumption levels, and estimated etiologic effects on cardiometabolic diseases

Dietary factor^a^	Educational level^b^	2012 intake among US adults^c^	Percent (%) change in intake per 10% price change^d^	Disease outcomes^e^	Unit of etiologic effect	Etiologic effect at age 50^f^	Etiologic effect at age 70^f^
		mean ± SD				RR (95% CIs)	RR (95% CIs)
Fruit (g/d) (excl. 100% fruit juices)	< HS	90.9 ± 88.3	15.5	↓ CHD	per 1 serving (100 g)/d	0.93 (0.89–0.97)	0.95 (0.92–0.98)
	HS	103 ± 105	14.2	↓ Ischemic stroke		0.86 (0.80–0.92)	0.90 (0.86–0.94)
	COL	146 ± 112	13.1	↓ Hemorrhagic stroke		0.69 (0.56–0.84)	0.77 (0.67–0.89)
Vegetables (g/d) (incl. legumes)	< HS	162 ± 79.6	15.5	↓ CHD	per 1 serving (100 g)/d	0.94 (0.91–0.97)	0.96 (0.94–0.98)
	HS	168 ± 92.1	14.2	↓ Ischemic stroke		0.80 (0.70–0.92)	0.91 (0.84–0.97)
	COL	217 ± 129	13.1	↓ Hemorrhagic stroke		0.80 (0.67–0.96)	0.86 (0.76–0.97)
Nuts/seeds (g/d)	< HS	5.78 ± 14.2	15.5	↓ CHD	per 1 serving (1 oz)/wk	0.91 (0.87–0.94)	0.93 (0.91–0.96)
	HS	9.87 ± 13.1	14.2	↓ Diabetes		0.96 (0.94–0.98)	0.97 (0.96–0.99)
	COL	17.7 ± 30.9	13.1				
Whole grains (g/d)	< HS	15.5 ± 16.1	15.5	↓ CHD	per 1 serving (50 g)/d	0.96 (0.93–0.99)	0.97 (0.95–0.99)
	HS	19.7 ± 17.9	14.2	↓ Ischemic stroke		0.90 (0.83–0.97)	0.93 (0.88–0.98)
	COL	26.5 ± 19.7	13.1	↓ Hemorrhagic stroke		0.90 (0.83–0.97)	0.93 (0.88–0.98)
				↓ Diabetes		0.86 (0.80–0.92)	0.90 (0.86–0.94)
Processed meats (g/d)	< HS	29.4 ± 14.1	–3.4	↑ CHD	per 1 serving (50 g)/d	1.24 (1.04–1.47)	1.16 (1.03–1.30)
	HS	33.6 ± 21.3	–3.2	↑ Diabetes		1.65 (1.30–2.08)	1.41 (1.20–1.65)
	COL	27.2 ± 16.5	–2.9				
Red meats, unprocessed (g/d)	< HS	52.8 ± 28.6	–3.4	↑ Diabetes	per 1 serving (100 g)/d	1.47 (1.14–1.88)	1.30 (1.09–1.54)
	HS	50.2 ± 19.7	–3.2				
	COL	40.0 ± 24.6	–2.9				
Sugar-sweetened beverages (8 oz/d)	< HS	1.49 ± 1.56	–7.3	↑ BMI (baseline BMI < 25)	↑ BMI (baseline BMI < 25)	0.10 kg/m^2^ (0.05–0.15)	0.10 kg/m^2^ (0.05–0.15)
	HS	1.31 ± 1.56	–6.7	↑ BMI (baseline BMI ≥ 25)		0.23 kg/m^2^ (0.14–0.32)	0.23 kg/m^2^ (0.14–0.32)
	COL	0.69 ± 0.99	–5.6	↑ CHD, direct effect (BMI adjusted)		1.26 (1.15–1.37)	1.17 (1.10–1.24)
				↑ Diabetes, direct effect (BMI adjusted)		1.27 (1.11–1.46)	1.18 (1.07–1.29)

^a^Dietary factors for which we identified probable or convincing evidence for etiologic effects on cardiometabolic diseases (CMD), including coronary heart disease (CHD), stroke, or type 2 diabetes mellitus; see text for further details

^b^Education strata were defined as less than high school education (< HS), high school or some college (HS), college graduates (COL)

^c^Based on nationally representative data combining the 2009–2010 and 2011–2012 cycles of the National Health and Nutrition Examination Survey for the adult US population (age 25+ years; N = 8516), accounting for complex survey design and sampling weights as appropriate [50]. Mean and SD of dietary intakes were estimated using two non-consecutive 24-hour dietary recalls per person; accounting for within-person variation and adjusting for total energy using the residual method (2000 kcal/d) to reduce measurement error and further account for differences in body size, metabolic efficiency, and physical activity. Intakes of food groups were obtained using the Food Patterns Equivalents Database, with servings converted to g/day [16]. Definitions and units for each dietary factor were defined to be consistent with definitions used in epidemiological studies or trials that provided evidence on etiologic effects on cardiometabolic diseases [51]

^d^The estimated percent change in the quantity of a food consumed in relation a percent change in its price, based on meta-analysis of prospective changes in intakes in response to changes in price of demand [21]. Estimated price responsiveness of healthy foods were based on findings for fruits and vegetables; and for unhealthy foods, on findings for sugar-sweetened beverages. We further accounted for differences in price-responsiveness by socioeconomic status based on a meta-analysis of global cross-sectional price-elasticity estimates (‘low gradient’ case, shown in this Table) and observed responses to a beverage excise tax in Mexico (‘high gradient’ case) [11, 23]; see text for further details

^e^Data on US deaths by age, sex, and education were derived from the National Center for Health Statistics, including deaths due to ischemic heart disease (ICD10: I20–I25), ischemic stroke (I63, I65–I67 (except I67.4), I69.3, G45), hemorrhagic and other non-ischemic stroke (I60–I62, I64, I69.0–I69.2, I69.4, I69.8, I67.4), diabetes mellitus (E10–E14, except E10.2, E11.2, E12.2, E13.2), and hypertensive heart disease (I11)

^f^Obtained from published or de novo dose-response meta-analyses of prospective cohorts or randomized trials. Meta-analyses were evaluated and selected based on design, number of studies and events, definitions of dietary exposure and disease outcomes, length of follow-up, statistical methods, evidence of bias, and control for confounders [18]. Estimated etiologic effects and uncertainty were quantified per standardized units. No differential effects on incidence versus case-specific mortality were identified for these dietary factors; thus, mortality effects were assumed to be similar to effects on incidence. For unclassified (other) strokes, we utilized the weighted average of effects (RRs) on ischemic and hemorrhagic stroke, based on the relative proportions of ischemic vs. hemorrhagic stroke deaths among classified cases in the National Center for Health Statistics database. We incorporated differences in proportional effects (RRs) by age in groups from 25–34 to 75+ years as previously established; representative RRs at age 50 and 70 are shown. We did not identify evidence for differences in etiologic effects by sex [18]

### Effect of price changes on dietary intakes

We obtained the impact of pricing changes on dietary intakes from a meta-analysis of prospective observational and interventional studies [21], allowing separate assessment of interventions to decrease versus those to increase prices. This meta-analysis provided estimates of the average own-price elasticity (the percentage change in intake in response to each 1% change in price) of –1.42 for fruits, vegetables, nuts/seeds, and whole grains, –0.32 for processed meats and red meats, and –0.73 for SSBs. We further assessed the literature demonstrating that price responsiveness varies with SES [8, 22]. We analyzed a ‘low gradient’ scenario, based on a meta-analysis that compared low- versus high-income households within different countries [23], which found an overall 18.2% higher price-responsiveness for low versus high SES groups (in this analysis, < HS and COL, respectively), as well as a ‘high gradient’ scenario, based on the empiric responsiveness to a 10% excise tax on SSBs in Mexico [11], which found a 65.4% price-responsiveness for low versus high SES groups. While individual cross-sectional US studies could be considered to model the price-responsiveness and variation by SES for different food groups [24–28], we chose not to favor estimates from cross-sectional or single studies, each of which could be hampered by their own individual limitations, as summarized by Lin et al. [28]. Rather, we utilized two comprehensive meta-analyses [21, 23] as well as recent empiric evidence in Mexico [11], and believe that the range of modelled variation between lower versus higher SES groups (from 18% to 65%) represents a reasonable range of potential differential effects by SES.

Finally, we considered a 10% price change in taxes or subsidies (increasing the price of unhealthy foods relative to healthy foods by approximately 20%) and, similarly, a higher scenario of a 30% price change. We modeled the direct effects of the price changes at the consumer level, rather than the specific policies required to attain them, in order to minimize assumptions in translating the latter to consumer prices.

### Mortality outcomes

The numbers of disease-specific deaths in 2012 by age, sex, and educational level were obtained from the National Center for Health Statistics Division of Vital Statistics (http://www.cdc.gov/nchs/deaths.htm). We excluded foreign residents (individuals who died in the US but who resided outside the US) and those with missing information on age (0.017% of deaths) or educational level (2.1%). We included deaths from coronary heart disease (ICD 10: I20–I25), ischemic stroke (I63, I65–I67 (except I67.4), I69.3, G45), hemorrhagic (I60–I62, I69.0–I69.2, I67.4), unidentified and other non-ischemic/hemorrhagic stroke (I64, I69.4, I69.8), diabetes mellitus (E10–E14, except E10.2, E11.2, E12.2, E13.2), and hypertensive heart disease (I11). For each stratum, the corresponding size of the US population by age, sex, and educational levle was estimated from the 2012 American Community Survey microdata sample including 2.15 million weighted records [29].

### Data analysis

We calculated the estimated CMD mortality preventable by changes in dietary intake in response to price changes using a comparative risk assessment framework [30]. Our analysis of etiologic effects incorporated direct effects of dietary changes on CMD mortality as well as BMI-mediated effects for SSBs. All models were stratified by age groups, sex, and education (< HS, HS, COL). In each stratum, the proportion of disease-specific mortality prevented by the intervention (potential impact fraction, PIF) was calculated using the following formula [31]:

$$ PIF=\frac{\int_{x=0}^m RR(x)P(x) dx-{\int}_{x=0}^m RR(x){P}^{\hbox{'}}(x) dx}{\int_{x=0}^m RR(x)P(x) dx} $$

where *RR(x)* is the RR at dietary intake level *x*, *P(x)* is the current stratum-specific distribution of dietary intake, *P’(x)* is the alternative stratum-specific distribution of dietary intake following the intervention, and *m* is the maximum dietary intake level.

In addition to the estimation of the effect of individual food targets, we also considered a potential joint effect, for example, as part of a tax-subsidy framework or similar incentive-disincentive system. Because summation of PIFs overestimates their joint effects, the joint impact for multiple dietary changes was estimated within each age-, sex-, and education-specific stratum based on the following formula:

$$ PI{F}_{joint}=1-\prod \limits_{r=1}^R\left(1- PI{F}_r\right) $$

where *PIF*_*joint*_ is the joint potential attributable fraction, *r* denotes each individual dietary factor, and *R* is the number of dietary factors. We recognized that certain dietary intakes may be correlated among individuals within each stratum, which could slightly overestimate the true joint effect. All inputs to the model were prepared using Stata SE version 14, College Station TX, and analyses were conducted using R version 3.1.0 [32]. A detailed description of the comparative risk assessment methodology is presented as a technical Appendix.

## Results

### Dietary factors, price-responsiveness, and etiologic effects

The selected dietary factors, current consumption levels, price-responsiveness, and estimated etiologic effects are shown in Table 1. The current intake of each dietary factor was far from the recommended intakes. Under the assumption of a low socioeconomic gradient in price-responsiveness, a 10% decrease in fruit prices would increase estimated consumption by 15.5% (14.1 g/d) among those with < HS education, 14.2% (14.6 g/d) among HS graduates, and 13.1% (19.1 g/d) among COL graduates. For SSBs, a 10% price increase would decrease estimated consumption by 7.3% (0.11 serv/d), 6.7% (0.08 serv/d), and 5.6% (0.03 serv/d), respectively. The estimated effects of such changes were generally quite modest; for example, a 14.1 g/d increase in fruit consumption was estimated to reduce CHD by 0.8% and a 0.11 serv/d reduction in SSBs was estimated to reduce diabetes by 2.5%.

### Cardiometabolic deaths potentially prevented by price changes

The estimated number of CMD deaths preventable by each price intervention are shown in Table 2. Jointly altering the prices of these seven dietary factors (10% each), and assuming a low SES gradient, was estimated to prevent 23,174 deaths/year (95% UI 22,024–24,595), corresponding to 3.4% (95% UI 3.2–3.6) of all CMD deaths in the US. A larger (30%) price change in all seven dietary targets was estimated to prevent approximately 63,268 deaths/year (95% UI 60,425–66,719) or 9.2% (95% UI 8.8–9.7) of all CMD deaths. By disease outcome, the largest proportional reductions were observed for stroke, followed by diabetes and CHD. Findings for stroke subtypes (ischemic, hemorrhagic, other) are shown in Additional file 1: Tables S1 and S2. Among individual dietary factors, the greatest estimated impact was for reducing the price of vegetables (6294 fewer CMD deaths/year), fruits (5265), and nuts/seeds (3413), and increasing the price of SSBs (4647). By cause, diabetes deaths would be most influenced by price changes in SSBs (1.5% reduction in deaths) and processed meats (0.7% reduction); while CHD deaths would be most influenced by price changes in SSBs (1% reduction) and nuts/seeds (0.9% reduction). The largest effect was observed for stroke by subsidizing fruits and vegetables (2.4% and 2.8% reduction, respectively).

**Table 2:** Annual cardiometabolic deaths potentially prevented by a 10% or 30% price change for selected foods in the US^a^

Dietary factors	Disease outcome^b^	10% price change^c^		30% price change^c^	
		No. of deaths/year prevented (95% UI)	Proportion (%) of deaths prevented (95% UI)	No. of deaths/year prevented (95% UI)	Proportion (%) of deaths prevented (95% UI)
Overall diet	CHD	12,236 (11,320–13,230)	3.4 (3.1–3.6)	33,293 (30,887–35,798)	9.2 (8.5–9.9)
	Hypertensive HD	45 (34–60)	0.1 (0.1–0.2)	134 (102–179)	0.4 (0.3–0.5)
	Stroke	6942 (6456–7430)	5.5 (5.1–5.9)	18726 (17,485–19,955)	14.9 (13.9–15.8)
	Diabetes	2274 (2063–2626)	3.4 (3.1–4.0)	6287 (5756–7050)	9.5 (8.7–10.6)
	CMD, total	23174 (22,024–24,595)	3.4 (3.2–3.6)	63268 (60,425–66,719)	9.2 (8.8–9.7)
Fruit	CHD	2213 (1852–2643)	0.6 (0.5–0.7)	6143 (5144–7316)	1.7 (1.4–2.0)
	Stroke	3038 (2726–3397)	2.4 (2.2–2.7)	8308 (7478–9256)	6.6 (5.9–7.4)
	CMD, total	5265 (4771–5817)	0.8 (0.7–0.8)	14475 (13125–15,974)	2.1 (1.9–2.3)
Vegetables	CHD	2873 (2443–3359)	0.8 (0.7–0.9)	8223 (7011–9578)	2.3 (1.9–2.6)
	Stroke	3423 (3044–3818)	2.7 (2.4–3.0)	9554 (8497–10,585)	7.6 (6.7–8.4)
	CMD, total	6294 (5722–6901)	0.9 (0.8–1.0)	17749 (16,176–19,458)	2.6 (2.4–2.8)
Nuts/seeds	CHD	3148 (2710–3599)	0.9 (0.7–1.0)	8214 (7116–9326)	2.3 (2.0–2.6)
	Diabetes	269 (227–316)	0.4 (0.3–0.5)	701 (592–822)	1.1 (0.9–1.2)
	CMD, total	3413 (2976–3863)	0.5 (0.4–0.6)	8912 (7788–10,049)	1.3 (1.1–1.5)
Whole grains	CHD	587 (457–720)	0.2 (0.1–0.2)	1741 (1356–2137)	0.5 (0.4–0.6)
	Stroke	514 (453–579)	0.4 (0.4–0.5)	1522 (1343–1712)	1.2 (1.1–1.4)
	Diabetes	425 (372–480)	0.6 (0.6–0.7)	1252 (1099–1413)	1.9 (1.7–2.1)
	CMD, total	1527 (1376–1683)	0.2 (0.2–0.2)	4518 (4072–4977)	0.7 (0.6–0.7)
Processed meats	CHD	1700 (1315–2206)	0.5 (0.4–0.6)	5048 (3906–6493)	1.4 (1.1–1.8)
	Diabetes	477 (385–580)	0.7 (0.6–0.9)	1408 (1141–1703)	2.1 (1.7–2.6)
	CMD, total	2175 (1777–2689)	0.3 (0.3–0.4)	6447 (5286–7944)	0.9 (0.8–1.2)
Red meats, unprocessed	Diabetes	140 (106–176)	0.2 (0.2–0.3)	419 (316–524)	0.6 (0.5–0.8)
Sugar sweetened beverages	CHD	3544 (2921–4302)	1.0 (0.8–1.2)	10091 (8354–12,027)	2.8 (2.3–3.3)
	Hypertensive HD	45 (34–60)	0.1 (0.1–0.2)	134 (102–179)	0.4 (0.3–0.5)
	Stroke	67 (60–76)	0.1 (0.0–0.1)	201 (178–226)	0.2 (0.1–0.2)
	Diabetes	986 (804–1349)	1.5 (1.2–2.0)	2729 (2267–3424)	4.1 (3.4–5.2)
	CMD, total	4647 (3993–5680)	0.7 (0.6–0.8)	13169 (11,428–15,366)	1.9 (1.7–2.2)

^a^Estimated using nationally representative data from the US adult population in 2012 based on a comparative risk assessment framework

^b^CVD corresponds to the sum of CHD, hypertensive heart disease, and stroke; CMD corresponds to the sum of CVD and diabetes. Values may not precisely add up due to rounding

^c^Estimates based on a low SES gradient (18.2% differential effect comparing those with lower than high school versus college education)

*CMD* cardiometabolic diseases, *CHD* coronary heart disease, *HD* heart disease, *UI* uncertainty interval

**Fig. 1:**
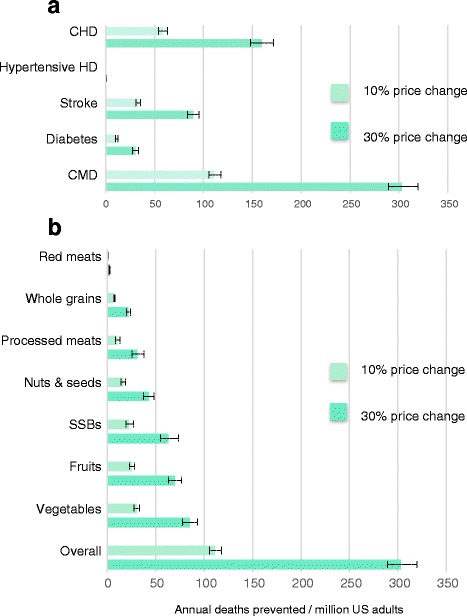
Annual US cardiometabolic deaths potentially prevented by a 10% or 30% price change in seven dietary targets. **a** Effects of price changes in all seven dietary targets, by cause. **b** Effects of price changes on total cardiometabolic deaths, by dietary target

Per year, 111 CMD deaths per million US adults could be potentially prevented by a 10% price change in all seven dietary targets, whereas 303 deaths/million could be potentially prevented by a 30% price change (Fig. 1). By cause, the largest impacts were seen for CHD (10% price change: 58 fewer CMD deaths/million; 30% price change: 159 fewer CMD deaths/million), followed by stroke (33 and 89 deaths/million, respectively) and diabetes (10 and 30 deaths/million, respectively). By dietary targets, the smallest effects on CMD mortality were estimated from altering the price of unprocessed red meat, whole grains, and processed meat, although with a 30% price change, the estimated benefits of altering whole grain and processed meat prices were notable.

### Disparities in cardiometabolic deaths potentially prevented by price changes

When stratified by educational attainment, a generally larger estimated proportion of CMD deaths would be prevented among those with < HS and HS, compared to those with COL (Table 3). Under the scenario of a low SES gradient in price responsiveness, a joint 10% price change would avert approximately 3.1% of CMD deaths among those with < HS, 3.6% among those with HS, and 2.9% among those with COL. Applying a higher SES gradient in price-responsiveness, the corresponding estimated reductions were 3.7%, 3.6%, and 2.5%, respectively. With a 30% price change, the corresponding estimated reductions were 10%, 9.8%, and 6.7%. Findings stratified by age and sex are presented in Additional file 1: Table S3.

**Table 3:** Annual cardiometabolic deaths potentially prevented by a 10% and 30% price change in 7 selected foods in the US, by educational attainment^a^

Disease outcome^b^	Price change scenario		< High school (n = 60,742,522)		High school (n = 119,506,708)^d^		College (n = 28,482,268)	
	% price change	SES gradient^c^	No. of deaths/year prevented (95% UI)	Proportion (%) of deaths prevented (95% UI)	No. of deaths/year prevented (95% UI)	Proportion (%) of deaths prevented (95% UI)	No. of deaths/year prevented (95% UI)	Proportion (%) of deaths prevented (95% UI)
CHD	10%	Low	2657 (2325–3008)	3.0 (2.6–3.4)	8054 (7255–8908)	3.7 (3.3–4.1)	1486 (1323–1728)	2.8 (2.5–3.2)
		High	3120 (2732–3531)	3.5 (3.1–3.9)	8054 (7255–8908)	3.7 (3.3–4.1)	1268 (1127–1478)	2.4 (2.1–2.8)
	30%	Low	7380 (6492–8243)	8.2 (7.3–9.2)	21816 (19755–23985)	9.9 (9.0–10.9)	3996 (3563–4560)	7.5 (6.7–8.5)
		High	8546 (7534–9532)	9.5 (8.4–10.6)	21816 (19755–23985)	9.9 (9.0–10.9)	3463 (3090–3960)	6.5 (5.8–7.4)
Hypertensive HD	10%	Low	12 (8–17)	0.1 (0.1–0.2)	29 (19–43)	0.1 (0.1–0.2)	3 (2–5)	0.1 (0.0–0.1)
		High	14 (10–21)	0.2 (0.1–0.3)	29 (19–43)	0.1 (0.1–0.2)	3 (2–4)	0.1 (0.0–0.1)
	30%	Low	35 (25–52)	0.4 (0.3–0.7)	87 (57–127)	0.4 (0.3–0.6)	10 (7–16)	0.2 (0.1–0.3)
		High	42 (29–61)	0.5 (0.4–0.8)	87 (57–127)	0.4 (0.3–0.6)	9 (6–13)	0.2 (0.1–0.3)
Stroke, total	10%	Low	1588 (1415–1767)	5.0 (4.5–5.6)	4307 (3871–4768)	5.8 (5.2–6.4)	1041 (939–1148)	5.4 (4.9–6.0)
		High	1864 (1662–2073)	5.9 (5.2–6.5)	4307 (3871–4768)	5.8 (5.2–6.4)	888 (801–981)	4.6 (4.2–5.1)
	30%	Low	4347 (3890–4803)	13.7 (12.3–15.1)	11633 (10518–12778)	15.5 (14–17.1)	2740 (2488–3002)	14.2 (12.9–15.6)
		High	5007 (4487–5524)	15.8 (14.1–17.4)	11633 (10518–12778)	15.5 (14–17.1)	2388 (2165–2620)	12.4 (11.2–13.6)
Diabetes	10%	Low	585 (498–697)	3.2 (2.7–3.8)	1488 (1297–1756)	3.7 (3.2–4.4)	197 (171–294)	2.4 (2.1–3.6)
		High	687 (586–815)	3.8 (3.2–4.5)	1488 (1297–1756)	3.7 (3.2–4.4)	167 (146–255)	2.0 (1.8–3.1)
	30%	Low	1631 (1410–1899)	9.0 (7.8–10.5)	4104 (3613–4700)	10.2 (9–11.7)	557 (489–705)	6.7 (5.9–8.5)
		High	1895 (1643–2202)	10.5 (9.1–12.2)	4104 (3613–4700)	10.2 (9–11.7)	476 (418–618)	5.8 (5.1–7.5)
CMD, total	10%	Low	5286 (4847–5735)	3.1 (2.9–3.4)	14954 (13897–16010)	3.6 (3.3–3.8)	2934 (2714–3557)	2.9 (2.7–3.5)
		High	6210 (5697–6735)	3.7 (3.4–4.0)	14954 (13897–16010)	3.6 (3.3–3.8)	2501 (2310–3074)	2.5 (2.3–3.0)
	30%	Low	14672 (13487–15843)	8.7 (8.0–9.3)	40732 (38042–43415)	9.8 (9.1–10.4)	7906 (7357–8853)	7.7 (7.2–8.7)
		High	16986 (15632–18331)	10 (9.2–10.8)	40732 (38042–43415)	9.8 (9.1–10.4)	6847 (6372–7751)	6.7 (6.2–7.6)

^a^Estimated using nationally representative data from the US adult population in 2012 based on a comparative risk assessment framework (fruits, vegetables, nuts/seeds, whole grains, processed meat, unprocessed red meat, and sugar-sweetened beverages)

^b^CVD corresponds to the sum of CHD, hypertensive heart disease and stroke, and CMD to the sum of CVD and diabetes. Values may not precisely sum due to rounding

^c^We evaluated two potential gradients by SES: a ‘low gradient’ scenario modeled based on a meta-analysis of price elasticity of food demand [23], reporting 18.2% greater responsiveness in the low vs. high SES groups, and a ‘high gradient’ scenario modeled after the differential responsiveness to SSBs taxation observed 1 year after the implementation of a 10% excise tax in Mexico [11], where a 65.4% greater responsiveness (low versus high SES) was noticed

^d^Those with average educational attainment are assumed to experience the average price-responsiveness, and thus estimates in this group are not influenced by a change in the gradient of responsiveness comparing lower vs. higher SES

*CMD* cardiometabolic diseases, *CHD* coronary heart disease, *HD* heart disease, *SES* socioeconomic status, *UI* uncertainty interval

**Fig. 2:**
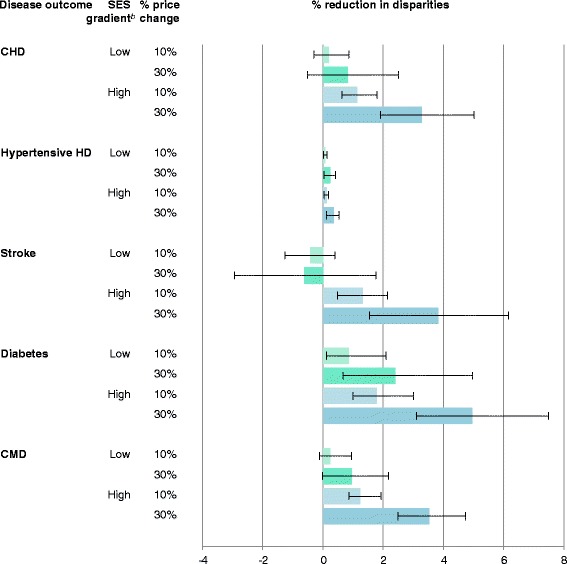
Reductions in disparities in US cardiometabolic mortality by 10% or 30% price change in seven dietary targets according to low and high price-responsiveness

Comparing those with < HS versus COL education and adjusting for population size (deaths per million adults), disparities in CMD deaths were evident (Additional file 1: Table S4). The relative reductions in CMD mortality disparities according to different pricing interventions are shown in Fig. 2. Under the scenario of a higher SES gradient in price-responsiveness, both 10% and 30% price changes in these seven dietary targets would reduce disparities in all outcomes. Under the scenario of a low SES gradient in price-responsiveness, these pricing interventions tended to reduce disparities for CHD, and total CMD, but not stroke, although none of these differences were statistically significant. In comparison, current disparities in diabetes mortality would be significantly reduced by any of the pricing scenarios.

## Discussion

By combining nationally representative datasets, we report, to our knowledge for the first time, the potential impact of strategies to alter food prices on CMD deaths in the US. A joint 10% price change was estimated to prevent 3.4% of all CMD deaths, while a larger price change (30%) was estimated to prevent 9.2% of all CMD deaths. None of the pricing scenarios significantly increased disparities; all would reduce disparities in diabetes deaths and, given a higher SES price-responsiveness, each would also reduce disparities in CHD, and stroke mortality. The largest impact was observed for decreasing prices of fruits and vegetables, and increasing the price of SSBs. By cause of death, reductions in stroke mortality were most effectively achieved by subsidies for vegetables and fruits, and in diabetes mortality by taxes on SSBs. These results are in line with previous modeling studies in South Africa and India, where a 20% SSB tax was estimated to reduce diabetes prevalence by 4% over 20 years [33, 34].

Many governments have already implemented fiscal measures to increase the price of unhealthy foods. One of the earliest was a 2011 Danish tax on saturated fat, later rescinded in 2013 due to controversy but estimated to have reduced national saturated fat intake by 4% [35]. Taxes exist in Hungary (on fat, sugar and energy drinks), Finland (sugar, expanding to soft drinks), Portugal (high salt products), and Mexico, France, and Latvia (SSBs) [36]. In Mexico, after the implementation of an 8% excise tax on non-essential foods (energy density > 275 kcal/100 g) and SSBs, the demand of these products decreased by approximately 5% from the predicted trend. Moreover, whereas no change was observed among high SES households, a 10% decrease was reported for those households of low SES [37]. South Africa and the UK also announced taxes on SSBs, effective in 2017 and 2018, respectively [38]. These efforts demonstrate the legal, practical, and political feasibility of food taxes. In the US, the cities of Berkeley and Philadelphia have passed excise taxes on SSBs [39], and state and national efforts have been deemed legally feasible [40]. Our findings suggest that the benefits of taxes for both health and disparities would be strongly complemented by accompanying strategies to reduce the price of fruits, vegetables, nuts, and whole grains. Subsidies are an essential component of a balanced pricing strategy to effectively improve diets, as well as to minimize the regressive nature of taxation alone [41]. Evidence from natural experiments and national interventions support this approach. In Finland, a combined strategy of agricultural, subsidy, and taxation policies resulted in an increased consumption of berries and a decreased consumption of animal fats versus increased consumption of vegetable oils, and a significant reduction of CVD risk factors and incidence [41, 42].

Given the growing inequities in diet and CMD in the US [16], our findings for disparities are particularly relevant. Since those with a lower SES have a lower intake of healthy foods, an intervention with a uniform proportional impact by SES would increase disparities; while higher intakes of healthy foods among those with a lower SES could lead to reduced disparities by uniformly effective interventions. Many types of interventions, such as education campaigns or food labeling, often have smaller effects among those with lower SES, potentially further exacerbating existing disparities [43]. In fact, most national efforts in the US focused on dietary education and guidelines/labeling improved overall dietary habits in all population subgroups, but much less among those with lower SES [16], finally resulting in increased dietary disparities over time.

Both nutrient- and food-specific taxes could be implemented by policy-makers. A recent report estimated that a sugar (nutrient) tax would have a larger impact on nutrition than a product-specific (SSB) tax, based on the broader base of products influenced by the former [44]. We focused this investigation on food-specific pricing changes based on the growing nutritional science, as highlighted by the 2015–2020 Dietary Guidelines for Americans [17], on the relevance of foods and overall diet patterns for health. Future studies should consider other pricing strategies, such as taxes on additives including added sugar and sodium.

We modeled final retail price changes of foods, which could be achieved by a range of potential strategies. Lower prices could be achieved by subsidies for agricultural practices, research and development or tax incentives for food manufacturers, retailers, and restaurants, or direct subsidies to wholesalers, retailers, or consumers [40]. In parallel, higher prices on certain foods could be achieved by changes in agricultural policies, tax disincentives or, most simply, by excise taxes [40]. Certain national US feeding programs, such as the Special Supplemental Nutrition Program for Women, Infants, and Children, already utilize such an approach by providing federal grants to states aiming to subsidize (by reducing the price through direct funding) nutritious foods for low-income, nutritionally at-risk populations [45]. At a broader scale, incentives for purchasing fruits and vegetables through rebates in the Supplemental Nutrition Assistance Program (SNAP) has proven successful to increase fruit and vegetable consumption and overall dietary quality in this population [46, 47]. Our findings suggest that a combination of financial incentives for fruits, vegetables, nuts, and whole grains (e.g., direct rebates, or increased relative purchasing power via the Electronic benefit transfer card), together with financial disincentives for SSBs and processed meats (e.g., via relative reductions in the purchasing power of the Electronic benefit transfer card for these food groups) would meaningfully reduce CMD and reduce disparities among SNAP participants. Given the large and growing inequities in dietary quality and CMD risk in the US [16], our findings for disparities are particularly relevant.

Our study has a number of strengths. We used nationally representative datasets on demographics, education, dietary habits, CVD risk factors (blood pressure and BMI), and cause-specific deaths, making our results generalizable to the US adult population. We limited our estimates of etiologic effects to a small number of specific dietary factors with the greatest evidence from meta-analyses, supported by consistency, dose-response, and plausible biology. Our model incorporates stratum-specific heterogeneity in underlying characteristics and intervention effects by age, sex, and education, increasing the validity of our results. We quantified uncertainty using Monte Carlo simulations, increasing interpretability and identifying the range of plausible effects.

Potential limitations should be considered. Dietary estimates were based on self-report, which could introduce errors into our estimates. We minimized this by using the average of two 24-h recalls per person and adjusting for both energy intake and within-person variation. The etiologic effect of each dietary factor on CMD was obtained from meta-analysis of mostly prospective observational data, which may be impacted by residual confounding (causing overestimation of effects) and by measurement error and regression dilution bias (causing underestimation of effects). However, these effects are supported by mechanistic evidence as well as a large randomized clinical trial that demonstrated reductions in CVD and diabetes highly comparable to the predicted effects from observational studies [4, 48]. While considerable evidence demonstrates the existence of differential price-responsiveness by SES, the precise magnitude of this gradient and how it might vary by underlying population characteristics are not perfectly characterized. We accounted for this by modeling both low and high gradients, providing findings for a range of plausible scenarios, and recognize that our results might modestly vary selecting alternative scenarios. In choosing our inputs for these scenarios, we favored estimates from meta-analyses and empirical national evidence over individual cross-sectional studies in the US, which show great variation in their estimates, to avoid favoring one particular study. We recognize that the efficacy of taxes on harmful foods will largely depend on what products consumers choose as an alternative. We did not incorporate specific substitution or complement effects (cross-price elasticity), which could potentially alter our results. This is especially relevant for price subsidies that have been found to be potentially counterproductive as they increase overall income to purchase food, including unhealthy products, when not applied in combination with unhealthy food taxes [49]; this supports our approach of combining subsidies and taxes. Additionally, we used meta-analysis of observed effects, incorporating average substitute and complement effects, as inputs to our model and the results most likely represent an average effect, which could be further augmented beyond our estimates by specifically encouraging or advocating for more healthful substitutes and complements. Furthermore, while approximately 80% of studies in our meta-analysis of interventional and prospective food pricing studies were from the US, the price elasticity estimates in the remaining countries were (non-statistically significantly) smaller. Thus, our findings may modestly underestimate the true health benefits of these food pricing strategies, compared to results based on US studies alone. Finally, given that effects of price changes on intake and of dietary changes on CMD are evident within 1 year [11, 48], we did not model lag-effects nor decay or acceleration of effects over time. Further research is needed to address how competing risks affect our mortality estimations and to incorporate the calculation of life years gained and reduction in disparities in life expectancy.

## Conclusion

Strategies introducing modest price changes on key dietary factors could reduce cardiovascular disease and diabetes burdens and disparities in the US. Policy-based strategies targeting disparities will require considering both baseline dietary habits as well as price responsiveness in specific population subgroups. The findings of our study have broad implications for policy-makers targeting fiscal measures to reduce CMD burden.

## Appendix

### PIF

While the formula presented in the paper is the standard description of the PIF used to communicate that comparative risk assessment is used to calculate attributable mortality, it gives only a vague insight as to how the PIF is calculated and what assumptions are being made. To clarify these assumptions, and to aid in reproducibility, we herein provide further details.

The PIF formula used is as follows:

$$ \frac{\int_{x=0}^m RR(x)P(x) dx-{\int}_{x=0}^m{RR}^{\hbox{'}}(x){P}^{\hbox{'}}(x) dx}{\int_{x=0}^m RR(x)P(x) dx} $$

Where $(x)$ is the distribution of current dietary consumption, (${P}^{\prime }(x)$) is the distribution of post-intervention dietary consumption, $RR(x)$ is the relative risk of mortality at exposure level $x$ pre-intervention, ${RR}^{\prime }(x)$ is the relative risk of mortality at exposure level $x$ post-intervention (price change), and $m$ is the maximum exposure level.

### $\boldsymbol{P}\left(\boldsymbol{x}\right)$ and ${\boldsymbol{P}}^{\prime}\left(\boldsymbol{x}\right)$

Current dietary intake follows a gamma distribution for all food and nutrient groups of interest. Previous research using comparative risk assessment in the field of nutrition has assumed intake follows a normal distribution or some variation thereof. However, NHANES data show that intake is right skewed for the food and nutrient groups of interest (in some cases, as with nuts, extremely so); therefore, we choose to assume intake follows a gamma distribution. Based on a visual inspection of histograms, we concluded that, overall, the gamma distribution fit the NHANES data better than an alternative right-skewed distribution (the log-normal), particularly for food groups where the intake is highly skewed, such as nuts/seeds. Simulations done to compare attributable mortality estimates assuming gamma, normal, and log-normal distributions to mortality estimates based on a non-parametric approach showed that estimates assuming the gamma distribution gave closer estimates to the non-parametric approach than the others.

Because the mean and variance of the gamma distribution is a function of the parameters of the gamma distribution ($E\left[X\right]=\frac{\alpha }{\beta }$, $Var\left[X\right]=\frac{\alpha }{\beta^2}$ where $X$ is a gamma random variable, $\alpha$ is the shape parameter and $\beta$ is the scale paraemter), estimates for the gamma parameter can be obtained from mean and variance estimates that account for survey design characteristics.

We assume post-intervention dietary consumption also follows a gamma distribution with mean and standard deviation of the current distribution multiplied by the intervention effect. Note that assuming each individual in the population experiences the same intervention effect would result in this post-intervention distribution.

### *RR*(*x*)

$RR(x)$ is defined to be

$$ \left\{\begin{array}{cc}\mathit{\exp}\left(\beta \left(x-y(x)\right)\right)& :x-y(x)\ge 0\\ {}1& :x-y(x)<0\end{array}\right. $$

where $\beta$ is the the change in log relative risk per unit of exposure, $x$ is the current exposure level, and $y(x)$ is the theoretical minimum risk exposure level. $y(x)$ is defined to be ${F}_{TMRED}\left({F}_X^{-1}(x)\right)$, where ${F}_{TMRED}$ is the cumulative distribution function of the theoretical minimum-risk exposure distribution (TMRED) and ${F}_X^{-1}$ is the inverse cumulative distribution function of the current exposure distribution. Similarly, ${RR}^{\prime }(x)$ is defined to be

$$ \left\{\begin{array}{cc}\mathit{\exp}\left(\beta \left(x-y(x)\right)\right)& :x-{y}^{\hbox{'}}(x)\ge 0\\ {}1& :x-{y}^{\hbox{'}}(x)<0\end{array}\right. $$

where $y(x)$ is defined to be ${F}_{TMRED}\left({F}_X{\prime}^{-1}(x)\right)$ and ${F}_X{\prime}^{-1}$ is the inverse cumulative distribution function of the counterfactual exposure.

Implicit in how we characterize the RR function are some of the fundamental assumptions we make about RR. Namely, that RR increases exponentially as distance from TMRED level ($y$) increases, that there is no risk attributable to exposure beyond the TMRED, and that both $x$ and TMRED level for an individual at exposure level $x$ are the $q$ -th quantile of their respective distributions (the observed exposure distribution/counterfactual exposure distribution and the TMRED, respectively). Note that the change in RR per unit of exposure is assumed to be the same pre- and post-intervention. $RR(x)$ and ${RR}^{\prime }(x)$ only differ because the TMRED level for an individual at exposure level $x$ differs.

### *m*

In our analyses, $m$ is defined to be $\infty$. Since the density of a gamma distribution approaches $0$ as exposure, $x$, approaches infinity, and because implausibly high values of exposure should exceed the corresponding theoretical maximum exposure level, implausibly high values of exposure will make no contributions to the PIF.

### Computation

In practice, we use simple numerical integration (using Riemann sums) to compute the integrals in the PIF formula. Thus, we use a categorical equivalent of the PIF formula

$$ PIF=\frac{\sum_{i=1}^n{P}_iR{R}_i-{\sum}_{i\hbox{'}=1}^nP{\hbox{'}}_{i\hbox{'}} RR{\hbox{'}}_{\hbox{'}i}}{\sum_{i\hbox{'}=1}^n{P}_iR{R}_i} $$

where the $n$ categories are determined by dividing up the exposure range (chosen here to be $\left[0,{F}_X^{-1}\left(\varPhi (6)\right)\right]$ for current exposure and $\left[0,{F}_{X\prime}^{-1}\left(\varPhi (6)\right)\right]$ for counterfactual exposure) into 121 intervals, each of length 0.1 when converted to the standard normal scale (except for the first one). More precisely, the range of exposure group $i$ can be described as follows:

$$ {\displaystyle \begin{array}{cc}\left[0,{F}_X^{-1}\left(\varPhi \left(-6\right)\right)\right]& :i=1\\ {}\left({F}_X^{-1}\left(\varPhi \left(-6+0.1\left({i}^{\prime }-2\right)\right)\right),{F}_X^{-1}\left(\varPhi \left(-6+0.1\left(i-1\right)\right)\right)\right]& :i>1\end{array}} $$

and for exposure group ${i}^{\prime }$:

$$ {\displaystyle \begin{array}{cc}\left[0,{F}_{X^{\prime}}^{-1}\left(\varPhi \left(-6\right)\right)\right]& :{i}^{\prime }=1\\ {}\left({F}_{X^{\prime}}^{-1}\left(\varPhi \left(-6+0.1\left({i}^{\prime }-2\right)\right)\right),{F}_{X^{\prime}}^{-1}\left(\varPhi \left(-6+0.1\left({i}^{\prime }-1\right)\right)\right)\right]& :{i}^{\prime }>1\end{array}} $$

### Monte Carlo simulations

Monte Carlo simulations were used to quantify uncertainty in the PIFs, incorporating uncertainty of estimates of exposure means, etiologic RRs, and intervention effects. Specifically, for each diet disease pair and stratum, we randomly drew 1000 times from the normal distribution of the estimate of disease-specific change in the log(RR) corresponding to a one unit increase in intake, the normal distribution of the estimate of the exposure mean, and the normal distribution of the estimate of the intervention effect. Draws of mean intake that were zero or less were changed to 0.00001. Each set of random draws was used to calculate the PIFs and attributable mortality.

### PIF via mediated effects

We used available log(RR) per unit increase in metabolic factors believed to mediate the effect of food intake on risk of death from cardiometabolic disease. Specifically, BMI is believed to mediate the effect of sugar-sweetened beverages on risk of death from various cardiometabolic diseases. We estimated log(RR) per unit associated increase in exposure for SSBs by taking the log(RR) per unit associated increase in exposure for BMI and multiplying it by an estimate of the associated increase in BMI per one unit associated increase in SSBs; our estimate of the associated increase in BMI per unit increase in SSBs for a given subgroup is a weighted average of the effect on BMI for overweight (BMI ≥ 25) individuals and non-overweight (BMI < 25) individuals, with the weights determined by the prevalence of overweight for that subgroup. Additionally, direct relationships with CHD and diabetes (after adjustment for BMI) were also included.

## Additional file

Additional file 1: Supplemental online materials. (DOCX 189 kb)
