# Supplementary material for: The potential impact of food taxes and subsidies on cardiovascular disease and diabetes burden and disparities in the United States
Source: BMC Med. 2017 Nov 27;15:208. doi: 10.1186/s12916-017-0971-9 (PMC5702980; doi:10.1186/s12916-017-0971-9)
Supplement: Additional file 1: — Supplemental online materials. (DOCX 189 kb) [file 12916_2017_971_MOESM1_ESM.docx]

**Table S1. Education-stratified stroke-related deaths potentially prevented by a 10% or 30% price change in 7 selected foods in the US ^a^**

| **Disease outcome** | **Elasticity scenario** | | **< High school (n 60,742,522)** | | **High school (n 119,506,708) ^c^** | | **College (n 28,482,268)** | |
| --- | --- | --- | --- | --- | --- | --- | --- | --- |
|  | **% price change** | **SES gradient ^b^** | **Deaths (n, 95% UIs)** | **Proportional deaths (%PIF, 95% UIs)** | **Deaths (n, 95% UIs)** | **Proportional deaths**  **(%PIF, 95% UIs)** | **Deaths (n, 95% UIs)** | **Proportional deaths (%PIF, 95% UIs)** |
|  |  |  |  |  |  |  |  |  |
| Ischemic stroke | 10% | Low | 169 (136, 199) | 4.0 (3.3, 4.8) | 406 (340, 482) | 4.4 (3.7, 5.2) | 100 (82, 119) | 4.2 (3.4, 5.0) |
|  |  | High | 198 (160, 234) | 4.7 (3.8, 5.6) | 406 (340, 482) | 4.4 (3.7, 5.2) | 85 (70, 101) | 3.6 (2.9, 4.2) |
|  | 30% | Low | 467 (379, 549) | 11.2 (9.1, 13.1) | 1116 (936, 1318) | 12.1 (10.1, 14.2) | 270 (224, 319) | 11.3 (9.4, 13.3) |
|  |  | High | 540 (439, 633) | 12.9 (10.5, 15.1) | 1116 (936, 1318) | 12.1 (10.1, 14.2) | 234 (194, 276) | 9.8 (8.1, 11.5) |
| Hemorrhagic stroke | 10% | Low | 439 (368, 515) | 6.4 (5.3, 7.5) | 1399 (1206, 1622) | 7.1 (6.1, 8.2) | 374 (316, 432) | 6.9 (5.8, 8.0) |
|  |  | High | 515 (431, 603) | 7.5 (6.3, 8.8) | 1399 (1206, 1622) | 7.1 (6.1, 8.2) | 319 (271, 370) | 5.9 (5.0, 6.8) |
|  | 30% | Low | 1183 (997, 1370) | 17.2 (14.5, 19.9) | 3718 (3232, 4263) | 18.9 (16.4, 21.6) | 965 (820, 1103) | 17.8 (15.1, 20.4) |
|  |  | High | 1357 (1146, 1569) | 19.7 (16.7, 22.8) | 3718 (3232, 4263) | 18.9 (16.4, 21.6) | 844 (717, 969) | 15.6 (13.2, 17.9) |
| Other stroke | 10% | Low | 980 (824, 1139) | 4.7 (4.0, 5.5) | 2504 (2140, 2895) | 5.5 (4.7, 6.3) | 567 (479, 660) | 4.9 (4.2, 5.8) |
|  |  | High | 1150 (968, 1337) | 5.6 (4.7, 6.5) | 2504 (2140, 2895) | 5.5 (4.7, 6.3) | 484 (408, 563) | 4.2 (3.6, 4.9) |
|  | 30% | Low | 2693 (2282, 3110) | 13.0 (11.0, 15.0) | 6788 (5843, 7768) | 14.8 (12.7, 16.9) | 1507 (1287, 1739) | 13.2 (11.2, 15.2) |
|  |  | High | 3106 (2639, 3581) | 15.0 (12.8, 17.3) | 6788 (5843, 7768) | 14.8 (12.7, 16.9) | 1311 (1116, 1517) | 11.4 (9.7, 13.2) |

^a^ Estimated using nationally representative data from the US adult population in 2012 based on a comparative risk assessment framework (fruits, vegetables, nuts/seeds, whole grains, processed meat, unprocessed red meat, and sugar-sweetened beverages).

^b^ We evaluated two potential gradients by SES: A “low gradient” scenario modeled based on a meta-analysis of price elasticity of food demand, [23] reporting18.2% greater responsiveness in the low vs high SES groups, and a “high gradient” scenario modeled after the differential responsiveness to SSBs taxation observed one year after the implementation of a 10% excise tax in Mexico, [11] where a 65.4% greater responsiveness (low versus high SES) was noticed.

^c^ Those with average educational attainment are assumed to experience the average price-responsiveness, and thus estimates in this group are not influenced by a change in the gradient of responsiveness comparing lower vs. higher socioeconomic status.

**Table S2. Education-stratified total and disease-specific cardiometabolic deaths potentially prevented by a 10% or 30% price change in all selected foods in the US ^a^**

|  |  |  | |  | **< High school (n 60,742,522)** | |  | **High school (n 119,506,708)** ^d^ | |  | **College (n 28,482,268)** | |
| --- | --- | --- | --- | --- | --- | --- | --- | --- | --- | --- | --- | --- |
| **Dietary factor** | **Disease outcome** ^b^ | **Price change scenario** ^c^ | |  | **No. of deaths/year prevented (95% UI)** | **Proportion (%) of deaths prevented (95% UI)** |  | **No. of deaths/year prevented (95% UI)** | **Proportion (%) of deaths prevented (95% UI)** |  | **No. of deaths/year prevented (95% UI)** | **Proportion (%) of deaths prevented (95% UI)** |
|  |  | **% price change** | **SES gradient** |  |  |  |  |  |  |  |  |  |
|  |  |  |  |  |  |  |  |  |  |  |  |  |
| **Fruits** | CHD | 10% | Low |  | 469 (353, 603) | 0.5 (0.4, 0.7) |  | 1431 (1083, 1829) | 0.7 (0.5, 0.8) |  | 315 (235, 393) | 0.6 (0.4, 0.7) |
|  |  |  | High |  | 552 (416, 710) | 0.6 (0.5, 0.8) |  | 1431 (1083, 1829) | 0.7 (0.5, 0.8) |  | 269 (201, 336) | 0.5 (0.4, 0.6) |
|  |  | 30% | Low |  | 1327 (1003, 1702) | 1.5 (1.1, 1.9) |  | 4007 (3038, 5121) | 1.8 (1.4, 2.3) |  | 824 (625, 1019) | 1.5 (1.2, 1.9) |
|  |  |  | High |  | 1541 (1168, 1975) | 1.7 (1.3, 2.2) |  | 4007 (3038, 5121) | 1.8 (1.4, 2.3) |  | 718 (543, 890) | 1.3 (1.0, 1.7) |
|  | Ischemic | 10% | Low |  | 45 (34, 57) | 1.1 (0.8, 1.4) |  | 127 (99, 159) | 1.4 (1.1, 1.7) |  | 27 (22, 33) | 1.1 (0.9, 1.4) |
|  | stroke |  | High |  | 53 (40, 68) | 1.3 (1.0, 1.6) |  | 127 (99, 159) | 1.4 (1.1, 1.7) |  | 23 (18, 28) | 1.0 (0.8, 1.2) |
|  |  | 30% | Low |  | 127 (96, 160) | 3.0 (2.3, 3.8) |  | 354 (275, 440) | 3.8 (3.0, 4.8) |  | 70 (57, 85) | 2.9 (2.4, 3.5) |
|  |  |  | High |  | 147 (112, 186) | 3.5 (2.7, 4.4) |  | 354 (275, 440) | 3.8 (3.0, 4.8) |  | 61 (50, 74) | 2.6 (2.1, 3.1) |
|  | Hemorrhagic | 10% | Low |  | 198 (155, 240) | 2.9 (2.3, 3.5) |  | 684 (553, 827) | 3.5 (2.8, 4.2) |  | 173 (140, 206) | 3.2 (2.6, 3.8) |
|  | stroke |  | High |  | 232 (182, 281) | 3.4 (2.6, 4.1) |  | 684 (553, 827) | 3.5 (2.8, 4.2) |  | 148 (119, 176) | 2.7 (2.2, 3.3) |
|  |  | 30% | Low |  | 545 (430, 656) | 7.9 (6.2, 9.5) |  | 1861 (1502, 2239) | 9.4 (7.6, 11.4) |  | 446 (365, 524) | 8.2 (6.7, 9.7) |
|  |  |  | High |  | 629 (497, 755) | 9.1 (7.2, 11) |  | 1861 (1502, 2239) | 9.4 (7.6, 11.4) |  | 391 (319, 459) | 7.2 (5.9, 8.5) |
|  | Other stroke | 10% | Low |  | 390 (302, 489) | 1.9 (1.5, 2.4) |  | 1158 (910, 1447) | 2.5 (2.0, 3.2) |  | 236 (186, 289) | 2.1 (1.6, 2.5) |
|  |  |  | High |  | 459 (356, 574) | 2.2 (1.7, 2.8) |  | 1158 (910, 1447) | 2.5 (2.0, 3.2) |  | 202 (159, 247) | 1.8 (1.4, 2.2) |
|  |  | 30% | Low |  | 1089 (851, 1353) | 5.3 (4.1, 6.5) |  | 3196 (2523, 3971) | 7.0 (5.5, 8.6) |  | 609 (489, 738) | 5.3 (4.3, 6.4) |
|  |  |  | High |  | 1261 (986, 1564) | 6.1 (4.8, 7.6) |  | 3196 (2523, 3971) | 7.0 (5.5, 8.6) |  | 532 (426, 646) | 4.6 (3.7, 5.6) |
|  | Stroke, total | 10% | Low |  | 631 (534, 745) | 2.0 (1.7, 2.3) |  | 1969 (1681, 2310) | 2.6 (2.2, 3.1) |  | 435 (382, 502) | 2.3 (2.0, 2.6) |
|  |  |  | High |  | 741 (628, 876) | 2.3 (2.0, 2.8) |  | 1969 (1681, 2310) | 2.6 (2.2, 3.1) |  | 372 (326, 430) | 1.9 (1.7, 2.2) |
|  |  | 30% | Low |  | 1755 (1486, 2063) | 5.5 (4.7, 6.5) |  | 5416 (4631, 6313) | 7.2 (6.2, 8.4) |  | 1126 (990, 1286) | 5.8 (5.1, 6.7) |
|  |  |  | High |  | 2031 (1723, 2382) | 6.4 (5.4, 7.5) |  | 5416 (4631, 6313) | 7.2 (6.2, 8.4) |  | 985 (865, 1124) | 5.1 (4.5, 5.8) |
|  | CMD, total | 10% | Low |  | 1098 (955, 1271) | 0.6 (0.6, 0.7) |  | 3405 (2930, 3929) | 0.8 (0.7, 0.9) |  | 752 (651, 852) | 0.7 (0.6, 0.8) |
|  |  |  | High |  | 1291 (1124, 1495) | 0.8 (0.7, 0.9) |  | 3405 (2930, 3929) | 0.8 (0.7, 0.9) |  | 643 (557, 728) | 0.6 (0.5, 0.7) |
|  |  | 30% | Low |  | 3076 (2688, 3556) | 1.8 (1.6, 2.1) |  | 9430 (8120, 10869) | 2.3 (2.0, 2.6) |  | 1952 (1693, 2198) | 1.9 (1.7, 2.2) |
|  |  |  | High |  | 3565 (3119, 4120) | 2.1 (1.8, 2.4) |  | 9430 (8120, 10869) | 2.3 (2.0, 2.6) |  | 1706 (1478, 1922) | 1.7 (1.4, 1.9) |
|  |  |  |  |  |  |  |  |  |  |  |  |  |
| **Vegetables** | CHD | 10% | Low |  | 684 (529, 852) | 0.8 (0.6, 1.0) |  | 1755 (1368, 2155) | 0.8 (0.6, 1.0) |  | 437 (335, 542) | 0.8 (0.6, 1.0) |
|  |  |  | High |  | 807 (623, 1004) | 0.9 (0.7, 1.1) |  | 1755 (1368, 2155) | 0.8 (0.6, 1.0) |  | 370 (284, 459) | 0.7 (0.5, 0.9) |
|  |  | 30% | Low |  | 1962 (1519, 2435) | 2.2 (1.7, 2.7) |  | 5032 (3937, 6153) | 2.3 (1.8, 2.8) |  | 1244 (953, 1539) | 2.3 (1.8, 2.9) |
|  |  |  | High |  | 2285 (1774, 2830) | 2.6 (2.0, 3.2) |  | 5032 (3937, 6153) | 2.3 (1.8, 2.8) |  | 1067 (817, 1324) | 2.0 (1.5, 2.5) |
|  | Ischemic | 10% | Low |  | 108 (77, 137) | 2.6 (1.9, 3.3) |  | 242 (181, 313) | 2.6 (2.0, 3.4) |  | 64 (48, 82) | 2.7 (2.0, 3.4) |
|  | stroke |  | High |  | 127 (91, 162) | 3.0 (2.2, 3.9) |  | 242 (181, 313) | 2.6 (2.0, 3.4) |  | 54 (40, 70) | 2.3 (1.7, 2.9) |
|  |  | 30% | Low |  | 303 (219, 384) | 7.2 (5.2, 9.2) |  | 676 (509, 865) | 7.3 (5.5, 9.3) |  | 177 (133, 226) | 7.4 (5.5, 9.5) |
|  |  |  | High |  | 351 (254, 444) | 8.4 (6.1, 10.6) |  | 676 (509, 865) | 7.3 (5.5, 9.3) |  | 153 (115, 195) | 6.4 (4.8, 8.2) |
|  | Hemorrhagic | 10% | Low |  | 215 (161, 279) | 3.1 (2.3, 4.0) |  | 635 (462, 805) | 3.2 (2.3, 4.1) |  | 179 (134, 230) | 3.3 (2.5, 4.2) |
|  | stroke |  | High |  | 253 (189, 327) | 3.7 (2.7, 4.8) |  | 635 (462, 805) | 3.2 (2.3, 4.1) |  | 152 (114, 196) | 2.8 (2.1, 3.6) |
|  |  | 30% | Low |  | 601 (448, 765) | 8.7 (6.5, 11.1) |  | 1756 (1288, 2199) | 8.9 (6.5, 11.2) |  | 488 (364, 621) | 9.0 (6.7, 11.5) |
|  |  |  | High |  | 696 (519, 884) | 10.1 (7.5, 12.8) |  | 1756 (1288, 2199) | 8.9 (6.5, 11.2) |  | 423 (315, 540) | 7.8 (5.8, 10.0) |
|  | Other stroke | 10% | Low |  | 520 (385, 659) | 2.5 (1.9, 3.2) |  | 1164 (874, 1480) | 2.5 (1.9, 3.2) |  | 289 (221, 375) | 2.5 (1.9, 3.3) |
|  |  |  | High |  | 612 (453, 775) | 3.0 (2.2, 3.7) |  | 1164 (874, 1480) | 2.5 (1.9, 3.2) |  | 245 (187, 318) | 2.1 (1.6, 2.8) |
|  |  | 30% | Low |  | 1466 (1095, 1846) | 7.1 (5.3, 8.9) |  | 3257 (2458, 4096) | 7.1 (5.4, 8.9) |  | 810 (619, 1043) | 7.1 (5.4, 9.1) |
|  |  |  | High |  | 1699 (1272, 2135) | 8.2 (6.2, 10.3) |  | 3257 (2458, 4096) | 7.1 (5.4, 8.9) |  | 697 (531, 900) | 6.1 (4.6, 7.9) |
|  | Stroke, total | 10% | Low |  | 842 (695, 996) | 2.7 (2.2, 3.1) |  | 2047 (1708, 2390) | 2.7 (2.3, 3.2) |  | 535 (444, 632) | 2.8 (2.3, 3.3) |
|  |  |  | High |  | 991 (819, 1172) | 3.1 (2.6, 3.7) |  | 2047 (1708, 2390) | 2.7 (2.3, 3.2) |  | 454 (377, 537) | 2.4 (2.0, 2.8) |
|  |  | 30% | Low |  | 2367 (1951, 2786) | 7.5 (6.1, 8.8) |  | 5704 (4761, 6637) | 7.6 (6.4, 8.9) |  | 1479 (1239, 1736) | 7.7 (6.4, 9.0) |
|  |  |  | High |  | 2744 (2265, 3220) | 8.6 (7.1, 10.1) |  | 5704 (4761, 6637) | 7.6 (6.4, 8.9) |  | 1277 (1066, 1506) | 6.6 (5.5, 7.8) |
|  | CMD, total | 10% | Low |  | 1527 (1315, 1748) | 0.9 (0.8, 1.0) |  | 3794 (3259, 4339) | 0.9 (0.8, 1.0) |  | 969 (835, 1116) | 0.9 (0.8, 1.1) |
|  |  |  | High |  | 1798 (1550, 2059) | 1.1 (0.9, 1.2) |  | 3794 (3259, 4339) | 0.9 (0.8, 1.0) |  | 822 (709, 947) | 0.8 (0.7, 0.9) |
|  |  | 30% | Low |  | 4328 (3739, 4947) | 2.6 (2.2, 2.9) |  | 10713 (9227, 12179) | 2.6 (2.2, 2.9) |  | 2718 (2336, 3124) | 2.7 (2.3, 3.1) |
|  |  |  | High |  | 5025 (4342, 5746) | 3.0 (2.6, 3.4) |  | 10713 (9227, 12179) | 2.6 (2.2, 2.9) |  | 2338 (2012, 2692) | 2.3 (2.0, 2.6) |
|  |  |  |  |  |  |  |  |  |  |  |  |  |
| **Nuts/seeds** | CHD | 10% | Low |  | 411 (297, 546) | 0.5 (0.3, 0.6) |  | 2340 (1920, 2763) | 1.1 (0.9, 1.3) |  | 394 (311, 480) | 0.7 (0.6, 0.9) |
|  |  |  | High |  | 481 (347, 639) | 0.5 (0.4, 0.7) |  | 2340 (1920, 2763) | 1.1 (0.9, 1.3) |  | 337 (267, 410) | 0.6 (0.5, 0.8) |
|  |  | 30% | Low |  | 1108 (801, 1465) | 1.2 (0.9, 1.6) |  | 6093 (5033, 7154) | 2.8 (2.3, 3.3) |  | 1006 (802, 1214) | 1.9 (1.5, 2.3) |
|  |  |  | High |  | 1276 (920, 1686) | 1.4 (1.0, 1.9) |  | 6093 (5033, 7154) | 2.8 (2.3, 3.3) |  | 880 (701, 1065) | 1.6 (1.3, 2.0) |
|  | Diabetes | 10% | Low |  | 39 (29, 51) | 0.2 (0.2, 0.3) |  | 202 (160, 248) | 0.5 (0.4, 0.6) |  | 28 (22, 35) | 0.3 (0.3, 0.4) |
|  |  |  | High |  | 46 (34, 60) | 0.3 (0.2, 0.3) |  | 202 (160, 248) | 0.5 (0.4, 0.6) |  | 24 (19, 30) | 0.3 (0.2, 0.4) |
|  |  | 30% | Low |  | 105 (78, 136) | 0.6 (0.4, 0.8) |  | 525 (419, 639) | 1.3 (1.0, 1.6) |  | 71 (56, 89) | 0.9 (0.7, 1.1) |
|  |  |  | High |  | 121 (89, 157) | 0.7 (0.5, 0.9) |  | 525 (419, 639) | 1.3 (1.0, 1.6) |  | 62 (49, 78) | 0.8 (0.6, 0.9) |
|  | CMD, total | 10% | Low |  | 451 (339, 585) | 0.3 (0.2, 0.3) |  | 2540 (2110, 2986) | 0.6 (0.5, 0.7) |  | 422 (339, 509) | 0.4 (0.3, 0.5) |
|  |  |  | High |  | 528 (396, 684) | 0.3 (0.2, 0.4) |  | 2540 (2110, 2986) | 0.6 (0.5, 0.7) |  | 361 (290, 436) | 0.4 (0.3, 0.4) |
|  |  | 30% | Low |  | 1219 (908, 1575) | 0.7 (0.5, 0.9) |  | 6619 (5536, 7702) | 1.6 (1.3, 1.9) |  | 1076 (873, 1286) | 1.1 (0.9, 1.3) |
|  |  |  | High |  | 1402 (1047, 1809) | 0.8 (0.6, 1.1) |  | 6619 (5536, 7702) | 1.6 (1.3, 1.9) |  | 942 (764, 1128) | 0.9 (0.7, 1.1) |
|  |  |  |  |  |  |  |  |  |  |  |  |  |
| **Whole grains** | CHD | 10% | Low |  | 121 (75, 169) | 0.1 (0.1, 0.2) |  | 377 (256, 497) | 0.2 (0.1, 0.2) |  | 88 (59, 120) | 0.2 (0.1, 0.2) |
|  |  |  | High |  | 143 (89, 200) | 0.2 (0.1, 0.2) |  | 377 (256, 497) | 0.2 (0.1, 0.2) |  | 74 (50, 101) | 0.1 (0.1, 0.2) |
|  |  | 30% | Low |  | 359 (223, 499) | 0.4 (0.2, 0.6) |  | 1119 (760, 1473) | 0.5 (0.3, 0.7) |  | 261 (176, 356) | 0.5 (0.3, 0.7) |
|  |  |  | High |  | 423 (263, 587) | 0.5 (0.3, 0.7) |  | 1119 (760, 1473) | 0.5 (0.3, 0.7) |  | 221 (149, 301) | 0.4 (0.3, 0.6) |
|  | Ischemic | 10% | Low |  | 15 (10, 21) | 0.4 (0.2, 0.5) |  | 38 (27, 51) | 0.4 (0.3, 0.5) |  | 10 (7, 13) | 0.4 (0.3, 0.6) |
|  | stroke |  | High |  | 18 (12, 25) | 0.4 (0.3, 0.6) |  | 38 (27, 51) | 0.4 (0.3, 0.5) |  | 8 (6, 11) | 0.3 (0.2, 0.5) |
|  |  | 30% | Low |  | 45 (30, 62) | 1.1 (0.7, 1.5) |  | 114 (79, 150) | 1.2 (0.9, 1.6) |  | 29 (21, 40) | 1.2 (0.9, 1.7) |
|  |  |  | High |  | 53 (36, 73) | 1.3 (0.8, 1.8) |  | 114 (79, 150) | 1.2 (0.9, 1.6) |  | 25 (18, 34) | 1.0 (0.7, 1.4) |
|  | Hemorrhagic | 10% | Low |  | 26 (19, 34) | 0.4 (0.3, 0.5) |  | 91 (70, 113) | 0.5 (0.4, 0.6) |  | 25 (20, 32) | 0.5 (0.4, 0.6) |
|  | stroke |  | High |  | 31 (23, 40) | 0.5 (0.3, 0.6) |  | 91 (70, 113) | 0.5 (0.4, 0.6) |  | 21 (17, 27) | 0.4 (0.3, 0.5) |
|  |  | 30% | Low |  | 78 (57, 100) | 1.1 (0.8, 1.5) |  | 269 (206, 333) | 1.4 (1.0, 1.7) |  | 75 (58, 95) | 1.4 (1.1, 1.8) |
|  |  |  | High |  | 91 (67, 118) | 1.3 (1.0, 1.7) |  | 269 (206, 333) | 1.4 (1.0, 1.7) |  | 64 (49, 80) | 1.2 (0.9, 1.5) |
|  | Other stroke | 10% | Low |  | 73 (54, 97) | 0.4 (0.3, 0.5) |  | 187 (143, 235) | 0.4 (0.3, 0.5) |  | 45 (33, 59) | 0.4 (0.3, 0.5) |
|  |  |  | High |  | 86 (63, 115) | 0.4 (0.3, 0.6) |  | 187 (143, 235) | 0.4 (0.3, 0.5) |  | 38 (28, 50) | 0.3 (0.2, 0.4) |
|  |  | 30% | Low |  | 215 (158, 285) | 1.0 (0.8, 1.4) |  | 554 (425, 697) | 1.2 (0.9, 1.5) |  | 134 (99, 175) | 1.2 (0.9, 1.5) |
|  |  |  | High |  | 253 (186, 335) | 1.2 (0.9, 1.6) |  | 554 (425, 697) | 1.2 (0.9, 1.5) |  | 114 (84, 148) | 1.0 (0.7, 1.3) |
|  | Stroke, total | 10% | Low |  | 115 (93, 140) | 0.4 (0.3, 0.4) |  | 317 (265, 372) | 0.4 (0.4, 0.5) |  | 81 (67, 96) | 0.4 (0.3, 0.5) |
|  |  |  | High |  | 136 (110, 166) | 0.4 (0.3, 0.5) |  | 317 (265, 372) | 0.4 (0.4, 0.5) |  | 68 (56, 81) | 0.4 (0.3, 0.4) |
|  |  | 30% | Low |  | 339 (275, 413) | 1.1 (0.9, 1.3) |  | 938 (786, 1101) | 1.3 (1.0, 1.5) |  | 239 (198, 283) | 1.2 (1.0, 1.5) |
|  |  |  | High |  | 398 (323, 486) | 1.3 (1.0, 1.5) |  | 938 (786, 1101) | 1.3 (1.0, 1.5) |  | 203 (168, 240) | 1.1 (0.9, 1.2) |
|  | Diabetes | 10% | Low |  | 97 (76, 118) | 0.5 (0.4, 0.7) |  | 273 (225, 325) | 0.7 (0.6, 0.8) |  | 56 (46, 68) | 0.7 (0.6, 0.8) |
|  |  |  | High |  | 115 (90, 139) | 0.6 (0.5, 0.8) |  | 273 (225, 325) | 0.7 (0.6, 0.8) |  | 47 (39, 58) | 0.6 (0.5, 0.7) |
|  |  | 30% | Low |  | 287 (225, 347) | 1.6 (1.2, 1.9) |  | 802 (662, 950) | 2.0 (1.7, 2.4) |  | 165 (136, 201) | 2.0 (1.6, 2.4) |
|  |  |  | High |  | 337 (265, 408) | 1.9 (1.5, 2.2) |  | 802 (662, 950) | 2.0 (1.7, 2.4) |  | 140 (116, 171) | 1.7 (1.4, 2.1) |
|  | CMD, total | 10% | Low |  | 333 (278, 393) | 0.2 (0.2, 0.2) |  | 965 (828, 1112) | 0.2 (0.2, 0.3) |  | 224 (192, 259) | 0.2 (0.2, 0.3) |
|  |  |  | High |  | 394 (329, 465) | 0.2 (0.2, 0.3) |  | 965 (828, 1112) | 0.2 (0.2, 0.3) |  | 190 (162, 219) | 0.2 (0.2, 0.2) |
|  |  | 30% | Low |  | 984 (822, 1161) | 0.6 (0.5, 0.7) |  | 2857 (2447, 3289) | 0.7 (0.6, 0.8) |  | 665 (568, 767) | 0.7 (0.6, 0.8) |
|  |  |  | High |  | 1158 (968, 1365) | 0.7 (0.6, 0.8) |  | 2857 (2447, 3289) | 0.7 (0.6, 0.8) |  | 564 (482, 650) | 0.6 (0.5, 0.6) |
|  |  |  |  |  |  |  |  |  |  |  |  |  |
| **Processed** | CHD | 10% | Low |  | 441 (291, 633) | 0.5 (0.3, 0.7) |  | 1049 (728, 1506) | 0.5 (0.3, 0.7) |  | 190 (126, 282) | 0.4 (0.2, 0.5) |
| **meats** |  |  | High |  | 521 (344, 748) | 0.6 (0.4, 0.8) |  | 1049 (728, 1506) | 0.5 (0.3, 0.7) |  | 161 (107, 238) | 0.3 (0.2, 0.4) |
|  |  | 30% | Low |  | 1308 (866, 1873) | 1.5 (1.0, 2.1) |  | 3111 (2168, 4455) | 1.4 (1.0, 2.0) |  | 567 (375, 838) | 1.1 (0.7, 1.6) |
|  |  |  | High |  | 1541 (1023, 2207) | 1.7 (1.1, 2.5) |  | 3111 (2168, 4455) | 1.4 (1.0, 2.0) |  | 480 (318, 709) | 0.9 (0.6, 1.3) |
|  | Diabetes | 10% | Low |  | 129 (95, 169) | 0.7 (0.5, 0.9) |  | 303 (221, 401) | 0.8 (0.6, 1.0) |  | 44 (31, 59) | 0.5 (0.4, 0.7) |
|  |  |  | High |  | 152 (112, 200) | 0.8 (0.6, 1.1) |  | 303 (221, 401) | 0.8 (0.6, 1.0) |  | 37 (26, 50) | 0.4 (0.3, 0.6) |
|  |  | 30% | Low |  | 382 (283, 499) | 2.1 (1.6, 2.8) |  | 895 (656, 1175) | 2.2 (1.6, 2.9) |  | 130 (93, 175) | 1.6 (1.1, 2.1) |
|  |  |  | High |  | 450 (333, 587) | 2.5 (1.8, 3.2) |  | 895 (656, 1175) | 2.2 (1.6, 2.9) |  | 110 (79, 148) | 1.3 (1.0, 1.8) |
|  | CMD, total | 10% | Low |  | 574 (413, 776) | 0.3 (0.2, 0.5) |  | 1356 (1017, 1825) | 0.3 (0.2, 0.4) |  | 234 (169, 323) | 0.2 (0.2, 0.3) |
|  |  |  | High |  | 678 (488, 917) | 0.4 (0.3, 0.5) |  | 1356 (1017, 1825) | 0.3 (0.2, 0.4) |  | 198 (143, 274) | 0.2 (0.1, 0.3) |
|  |  | 30% | Low |  | 1702 (1228, 2298) | 1.0 (0.7, 1.4) |  | 4017 (3017, 5397) | 1.0 (0.7, 1.3) |  | 698 (502, 961) | 0.7 (0.5, 0.9) |
|  |  |  | High |  | 2008 (1449, 2710) | 1.2 (0.9, 1.6) |  | 4017 (3017, 5397) | 1.0 (0.7, 1.3) |  | 591 (425, 814) | 0.6 (0.4, 0.8) |
|  |  |  |  |  |  |  |  |  |  |  |  |  |
| **Red meats,** | Diabetes | 10% | Low |  | 38 (25, 55) | 0.2 (0.1, 0.3) |  | 88 (57, 123) | 0.2 (0.1, 0.3) |  | 13 (9, 19) | 0.2 (0.1, 0.2) |
| **unprocessed** |  |  | High |  | 44 (30, 65) | 0.2 (0.2, 0.4) |  | 88 (57, 123) | 0.2 (0.1, 0.3) |  | 11 (7, 16) | 0.1 (0.1, 0.2) |
|  |  | 30% | Low |  | 112 (75, 163) | 0.6 (0.4, 0.9) |  | 263 (170, 366) | 0.7 (0.4, 0.9) |  | 39 (26, 56) | 0.5 (0.3, 0.7) |
|  |  |  | High |  | 132 (88, 193) | 0.7 (0.5, 1.1) |  | 263 (170, 366) | 0.7 (0.4, 0.9) |  | 33 (22, 48) | 0.4 (0.3, 0.6) |
|  |  |  |  |  |  |  |  |  |  |  |  |  |
| **Sugar-** | CHD | 10% | Low |  | 988 (760, 1252) | 1.1 (0.8, 1.4) |  | 2252 (1739, 2878) | 1.0 (0.8, 1.3) |  | 269 (195, 474) | 0.5 (0.4, 0.9) |
| **sweetened** |  |  | High |  | 1164 (896, 1473) | 1.3 (1.0, 1.6) |  | 2252 (1739, 2878) | 1.0 (0.8, 1.3) |  | 228 (165, 409) | 0.4 (0.3, 0.8) |
| **beverages** |  | 30% | Low |  | 2828 (2191, 3544) | 3.2 (2.4, 4.0) |  | 6431 (4982, 8112) | 2.9 (2.3, 3.7) |  | 781 (571, 1186) | 1.5 (1.1, 2.2) |
|  |  |  | High |  | 3301 (2567, 4132) | 3.7 (2.9, 4.6) |  | 6431 (4982, 8112) | 2.9 (2.3, 3.7) |  | 665 (485, 1039) | 1.2 (0.9, 1.9) |
|  | Hypertensive HD | 10% | Low |  | 12 (8, 17) | 0.1 (0.1, 0.2) |  | 29 (19, 43) | 0.1 (0.1, 0.2) |  | 3 (2, 5) | 0.1 (0, 0.1) |
|  |  |  | High |  | 14 (10, 21) | 0.2 (0.1, 0.3) |  | 29 (19, 43) | 0.1 (0.1, 0.2) |  | 3 (2, 4) | 0.1 (0, 0.1) |
|  |  | 30% | Low |  | 35 (25, 52) | 0.4 (0.3, 0.7) |  | 87 (57, 127) | 0.4 (0.3, 0.6) |  | 10 (7, 16) | 0.2 (0.1, 0.3) |
|  |  |  | High |  | 42 (29, 61) | 0.5 (0.4, 0.8) |  | 87 (57, 127) | 0.4 (0.3, 0.6) |  | 9 (6, 13) | 0.2 (0.1, 0.3) |
|  | Ischemic | 10% | Low |  | 2 (1, 2) | 0 (0, 0.1) |  | 4 (3, 5) | 0 (0, 0) |  | 0 (0, 1) | 0 (0, 0) |
|  | stroke |  | High |  | 2 (2, 3) | 0.1 (0, 0.1) |  | 4 (3, 5) | 0 (0, 0) |  | 0 (0, 1) | 0 (0, 0) |
|  |  | 30% | Low |  | 5 (4, 7) | 0.1 (0.1, 0.2) |  | 11 (8, 14) | 0.1 (0.1, 0.1) |  | 1 (1, 2) | 0.1 (0, 0.1) |
|  |  |  | High |  | 6 (5, 8) | 0.2 (0.1, 0.2) |  | 11 (8, 14) | 0.1 (0.1, 0.1) |  | 1 (1, 2) | 0 (0, 0.1) |
|  | Hemorrhagic | 10% | Low |  | 8 (6, 10) | 0.1 (0.1, 0.2) |  | 20 (15, 24) | 0.1 (0.1, 0.1) |  | 2 (2, 3) | 0 (0, 0.1) |
|  | stroke |  | High |  | 10 (7, 12) | 0.1 (0.1, 0.2) |  | 20 (15, 24) | 0.1 (0.1, 0.1) |  | 2 (1, 3) | 0 (0, 0) |
|  |  | 30% | Low |  | 24 (19, 31) | 0.4 (0.3, 0.5) |  | 59 (46, 73) | 0.3 (0.2, 0.4) |  | 7 (5, 9) | 0.1 (0.1, 0.2) |
|  |  |  | High |  | 29 (22, 37) | 0.4 (0.3, 0.5) |  | 59 (46, 73) | 0.3 (0.2, 0.4) |  | 6 (4, 8) | 0.1 (0.1, 0.1) |
|  | Other stroke | 10% | Low |  | 10 (7, 13) | 0 (0, 0.1) |  | 19 (14, 25) | 0 (0, 0.1) |  | 2 (2, 3) | 0 (0, 0) |
|  |  |  | High |  | 11 (8, 15) | 0.1 (0, 0.1) |  | 19 (14, 25) | 0 (0, 0.1) |  | 2 (1, 3) | 0 (0, 0) |
|  |  | 30% | Low |  | 29 (21, 39) | 0.1 (0.1, 0.2) |  | 56 (42, 75) | 0.1 (0.1, 0.2) |  | 7 (5, 9) | 0.1 (0, 0.1) |
|  |  |  | High |  | 34 (25, 46) | 0.2 (0.1, 0.2) |  | 56 (42, 75) | 0.1 (0.1, 0.2) |  | 6 (4, 8) | 0 (0, 0.1) |
|  | Stroke, total | 10% | Low |  | 20 (16, 24) | 0.1 (0.1, 0.1) |  | 42 (36, 50) | 0.1 (0, 0.1) |  | 5 (4, 6) | 0 (0, 0) |
|  |  |  | High |  | 23 (19, 28) | 0.1 (0.1, 0.1) |  | 42 (36, 50) | 0.1 (0, 0.1) |  | 4 (3, 5) | 0 (0, 0) |
|  |  | 30% | Low |  | 59 (49, 72) | 0.2 (0.2, 0.2) |  | 126 (107, 149) | 0.2 (0.1, 0.2) |  | 15 (12, 18) | 0.1 (0.1, 0.1) |
|  |  |  | High |  | 70 (58, 85) | 0.2 (0.2, 0.3) |  | 126 (107, 149) | 0.2 (0.1, 0.2) |  | 13 (10, 15) | 0.1 (0.1, 0.1) |
|  | Diabetes | 10% | Low |  | 286 (212, 386) | 1.6 (1.2, 2.1) |  | 638 (483, 880) | 1.6 (1.2, 2.2) |  | 56 (40, 153) | 0.7 (0.5, 1.8) |
|  |  |  | High |  | 336 (250, 451) | 1.9 (1.4, 2.5) |  | 638 (483, 880) | 1.6 (1.2, 2.2) |  | 48 (34, 135) | 0.6 (0.4, 1.6) |
|  |  | 30% | Low |  | 799 (601, 1019) | 4.4 (3.3, 5.6) |  | 1769 (1375, 2317) | 4.4 (3.4, 5.8) |  | 163 (116, 328) | 2.0 (1.4, 4.0) |
|  |  |  | High |  | 931 (703, 1179) | 5.1 (3.9, 6.5) |  | 1769 (1375, 2317) | 4.4 (3.4, 5.8) |  | 138 (99, 295) | 1.7 (1.2, 3.6) |
|  | CMD, total | 10% | Low |  | 1311 (1080, 1612) | 0.8 (0.6, 1.0) |  | 2974 (2415, 3645) | 0.7 (0.6, 0.9) |  | 338 (257, 981) | 0.3 (0.3, 1.0) |
|  |  |  | High |  | 1543 (1269, 1879) | 0.9 (0.7, 1.1) |  | 2974 (2415, 3645) | 0.7 (0.6, 0.9) |  | 286 (217, 878) | 0.3 (0.2, 0.9) |
|  |  | 30% | Low |  | 3721 (3060, 4527) | 2.2 (1.8, 2.7) |  | 8431 (6903, 10089) | 2.0 (1.7, 2.4) |  | 978 (755, 1793) | 1.0 (0.7, 1.8) |
|  |  |  | High |  | 4346 (3584, 5268) | 2.6 (2.1, 3.1) |  | 8431 (6903, 10089) | 2.0 (1.7, 2.4) |  | 832 (641, 1632) | 0.8 (0.6, 1.6) |

^a^ Estimated using nationally representative data from the US adult population in 2012 based on a comparative risk assessment framework (fruits, vegetables, nuts/seeds, whole grains, processed meat, unprocessed red meat, and sugar-sweetened beverages).

^b^ CVD corresponds to the sum of CHD, hypertensive heart disease and stroke; and CMD to the sum of CVD and diabetes. Values may not precisely sum due to rounding.

^c^ We evaluated two potential gradients by SES: A “low gradient” scenario modeled based on a meta-analysis of price elasticity of food demand, [23] reporting18.2% greater responsiveness in the low vs high SES groups, and a “high gradient” scenario modeled after the differential responsiveness to SSBs taxation observed one year after the implementation of a 10% excise tax in Mexico, [11] where a 65.4% greater responsiveness (low versus high SES) was noticed.

^d^ Those with average educational attainment are assumed to experience the average price-responsiveness, and thus estimates in this group are not influenced by a change in the gradient of responsiveness comparing lower vs. higher socioeconomic status.

**Table S3. Annual cardiometabolic deaths potentially prevented by a 10% price change in selected foods in the US, by age, sex, and educational attainment ^a^**

|  |  |  |  | **< High school (n 60,742,522)** | |  | **High school (n 119,506,708)** | |  | **College (n 28,482,268)** | |
| --- | --- | --- | --- | --- | --- | --- | --- | --- | --- | --- | --- |
| **Dietary factor** | **Disease outcome** ^b^ | **Gender** | **Age group** | **No. of deaths/year prevented (95% UI)** | **Proportion (%) of deaths prevented (95% UI)** |  | **No. of deaths/year prevented (95% UI)** | **Proportion (%) of deaths prevented (95% UI)** |  | **No. of deaths/year prevented (95% UI)** | **Proportion (%) of deaths prevented (95% UI)** |
|  |  |  |  |  |  |  |  |  |  |  |  |
| **Fruits** | CHD | Males | 25 | 2 (1, 3) | 0.9 (0.3, 1.7) |  | 5 (2, 9) | 1.0 (0.4, 1.8) |  | 1 (0, 1) | 1.0 (0.4, 1.7) |
|  |  |  | 35 | 5 (2, 10) | 0.7 (0.3, 1.3) |  | 19 (8, 33) | 0.7 (0.3, 1.2) |  | 5 (2, 9) | 1.2 (0.5, 2.1) |
|  |  |  | 45 | 22 (9, 40) | 0.7 (0.3, 1.2) |  | 113 (46, 202) | 1.0 (0.4, 1.8) |  | 17 (7, 30) | 0.9 (0.4, 1.6) |
|  |  |  | 55 | 54 (21, 94) | 0.9 (0.4, 1.6) |  | 168 (67, 289) | 0.8 (0.3, 1.3) |  | 48 (19, 80) | 0.9 (0.3, 1.5) |
|  |  |  | 65 | 50 (17, 93) | 0.6 (0.2, 1.0) |  | 153 (60, 260) | 0.6 (0.2, 1.1) |  | 62 (25, 108) | 0.9 (0.4, 1.5) |
|  |  |  | 75+ | 100 (45, 167) | 0.4 (0.2, 0.6) |  | 297 (139, 476) | 0.5 (0.2, 0.8) |  | 92 (41, 149) | 0.4 (0.2, 0.7) |
|  |  | Females | 25 | 0 (0, 1) | 0.7 (0.3, 1.2) |  | 2 (1, 3) | 1.0 (0.4, 1.7) |  | 0 (0, 1) | 1.4 (0.6, 2.4) |
|  |  |  | 35 | 3 (1, 6) | 1.0 (0.4, 1.9) |  | 7 (3, 13) | 0.8 (0.3, 1.4) |  | 2 (1, 3) | 1.4 (0.5, 2.3) |
|  |  |  | 45 | 11 (4, 19) | 0.9 (0.4, 1.6) |  | 36 (14, 61) | 1.0 (0.4, 1.6) |  | 5 (2, 8) | 1.0 (0.4, 1.7) |
|  |  |  | 55 | 23 (9, 41) | 0.9 (0.4, 1.7) |  | 72 (26, 125) | 0.8 (0.3, 1.4) |  | 14 (5, 23) | 0.9 (0.3, 1.4) |
|  |  |  | 65 | 35 (12, 61) | 0.7 (0.3, 1.3) |  | 114 (43, 189) | 0.8 (0.3, 1.4) |  | 18 (7, 29) | 0.8 (0.3, 1.3) |
|  |  |  | 75+ | 156 (78, 260) | 0.5 (0.2, 0.8) |  | 439 (215, 722) | 0.6 (0.3, 1.0) |  | 47 (21, 79) | 0.4 (0.2, 0.6) |
|  | Ischemic | Males | 25 | 0 (0, 0) | 1.9 (0.9, 3.4) |  | 0 (0, 1) | 2.1 (1.1, 3.4) |  | 0 (0, 0) | 2.0 (1.1, 3.2) |
|  | stroke |  | 35 | 0 (0, 1) | 1.5 (0.8, 2.5) |  | 1 (1, 2) | 1.6 (0.8, 2.5) |  | 0 (0, 0) | 2.6 (1.5, 4.0) |
|  |  |  | 45 | 1 (1, 2) | 1.4 (0.8, 2.3) |  | 5 (3, 8) | 2.2 (1.1, 3.6) |  | 1 (0, 1) | 2.0 (1.1, 3.1) |
|  |  |  | 55 | 3 (2, 5) | 1.9 (1.0, 3.1) |  | 7 (4, 11) | 1.6 (0.8, 2.6) |  | 2 (1, 3) | 1.9 (1.0, 2.9) |
|  |  |  | 65 | 3 (2, 5) | 1.2 (0.6, 2.0) |  | 8 (4, 12) | 1.3 (0.7, 2.1) |  | 4 (2, 6) | 2.0 (1.1, 2.9) |
|  |  |  | 75+ | 9 (5, 13) | 0.8 (0.4, 1.2) |  | 22 (13, 34) | 1.1 (0.7, 1.6) |  | 8 (5, 11) | 0.9 (0.5, 1.3) |
|  |  | Females | 25 | 0 (0, 0) | 1.4 (0.7, 2.3) |  | 0 (0, 1) | 2.1 (1.1, 3.2) |  | 0 (0, 0) | 2.9 (1.6, 4.4) |
|  |  |  | 35 | 0 (0, 0) | 2.1 (1.1, 3.6) |  | 1 (0, 1) | 1.7 (0.9, 2.7) |  | 1 (0, 1) | 3.0 (1.7, 4.7) |
|  |  |  | 45 | 1 (0, 1) | 1.9 (1.0, 3.0) |  | 3 (2, 5) | 2.0 (1.1, 3.1) |  | 1 (0, 1) | 2.1 (1.1, 3.2) |
|  |  |  | 55 | 2 (1, 4) | 2.0 (1.1, 3.2) |  | 5 (3, 8) | 1.7 (1.0, 2.7) |  | 1 (1, 2) | 1.9 (1.0, 2.8) |
|  |  |  | 65 | 4 (2, 6) | 1.5 (0.8, 2.4) |  | 11 (6, 17) | 1.8 (1.0, 2.8) |  | 2 (1, 2) | 1.6 (0.9, 2.5) |
|  |  |  | 75+ | 21 (12, 32) | 1.0 (0.6, 1.5) |  | 62 (34, 93) | 1.3 (0.7, 2.0) |  | 8 (4, 12) | 0.8 (0.4, 1.2) |
|  | Hemorrhagic | Males | 25 | 2 (1, 3) | 3.9 (1.7, 7.0) |  | 7 (3, 12) | 4.8 (2.2, 8.0) |  | 1 (0, 2) | 4.4 (2.1, 7.5) |
|  | stroke |  | 35 | 5 (3, 9) | 3.3 (1.6, 5.5) |  | 13 (6, 22) | 3.5 (1.7, 5.6) |  | 5 (2, 8) | 5.8 (2.5, 9.3) |
|  |  |  | 45 | 13 (6, 21) | 3.1 (1.4, 5.1) |  | 53 (23, 91) | 4.9 (2.2, 8.5) |  | 9 (4, 14) | 4.5 (2.0, 7.2) |
|  |  |  | 55 | 23 (10, 38) | 4.6 (2.1, 7.6) |  | 59 (25, 96) | 3.6 (1.6, 5.9) |  | 17 (8, 28) | 4.3 (2.0, 6.9) |
|  |  |  | 65 | 15 (7, 28) | 2.7 (1.2, 5.0) |  | 50 (24, 82) | 3.0 (1.4, 4.9) |  | 29 (12, 47) | 4.6 (1.9, 7.4) |
|  |  |  | 75+ | 27 (13, 44) | 1.8 (0.9, 3.0) |  | 95 (51, 148) | 2.6 (1.4, 4.1) |  | 35 (19, 54) | 2.1 (1.2, 3.3) |
|  |  | Females | 25 | 1 (0, 2) | 2.8 (1.2, 4.8) |  | 5 (2, 8) | 4.5 (2.4, 7.3) |  | 1 (1, 2) | 6.5 (3.2, 10.1) |
|  |  |  | 35 | 5 (2, 9) | 4.6 (2.2, 8.2) |  | 12 (6, 19) | 3.6 (1.8, 5.8) |  | 6 (3, 10) | 6.8 (2.9, 11.2) |
|  |  |  | 45 | 12 (5, 20) | 4.3 (2.0, 7.2) |  | 49 (25, 78) | 4.5 (2.3, 7.2) |  | 10 (4, 15) | 4.8 (2.1, 7.4) |
|  |  |  | 55 | 16 (7, 28) | 4.7 (2.0, 8.2) |  | 57 (24, 91) | 4.0 (1.7, 6.5) |  | 15 (6, 24) | 4.4 (1.9, 7.2) |
|  |  |  | 65 | 19 (9, 31) | 3.5 (1.6, 5.8) |  | 79 (36, 126) | 4.3 (1.9, 6.8) |  | 18 (8, 29) | 4.0 (1.8, 6.5) |
|  |  |  | 75+ | 57 (29, 90) | 2.3 (1.2, 3.6) |  | 199 (112, 303) | 3.1 (1.7, 4.7) |  | 26 (14, 41) | 1.9 (1.0, 3.0) |
|  | Other | Males | 25 | 0 (0, 1) | 3.7 (1.7, 6.7) |  | 1 (1, 2) | 4.3 (1.9, 7.1) |  | 0 (0, 0) | 4.1 (2.0, 6.7) |
|  | stroke |  | 35 | 1 (1, 2) | 3.2 (1.7, 5.4) |  | 5 (3, 8) | 3.2 (1.6, 4.9) |  | 1 (1, 2) | 5.5 (2.7, 8.9) |
|  |  |  | 45 | 7 (4, 12) | 2.8 (1.4, 4.7) |  | 29 (15, 47) | 4.5 (2.3, 7.3) |  | 4 (2, 7) | 4.2 (2.1, 6.7) |
|  |  |  | 55 | 25 (13, 41) | 3.8 (2.0, 6.1) |  | 61 (31, 96) | 3.2 (1.6, 5.1) |  | 16 (8, 24) | 3.8 (2.0, 5.8) |
|  |  |  | 65 | 31 (16, 53) | 2.3 (1.1, 3.8) |  | 80 (41, 126) | 2.5 (1.3, 4.0) |  | 34 (19, 53) | 3.9 (2.1, 5.9) |
|  |  |  | 75+ | 78 (44, 119) | 1.4 (0.8, 2.1) |  | 222 (130, 331) | 2.1 (1.2, 3.1) |  | 74 (42, 111) | 1.7 (0.9, 2.5) |
|  |  | Females | 25 | 0 (0, 0) | 2.6 (1.3, 4.7) |  | 1 (1, 2) | 4.1 (2.2, 6.4) |  | 0 (0, 1) | 5.3 (2.9, 7.8) |
|  |  |  | 35 | 2 (1, 3) | 4.4 (2.0, 7.7) |  | 4 (2, 6) | 3.4 (1.8, 5.3) |  | 1 (1, 2) | 6.0 (3.1, 9.5) |
|  |  |  | 45 | 7 (3, 11) | 3.9 (1.9, 6.3) |  | 20 (10, 32) | 4.2 (2.1, 6.5) |  | 3 (1, 5) | 4.4 (2.2, 7.0) |
|  |  |  | 55 | 17 (9, 28) | 4.0 (2.1, 6.7) |  | 51 (26, 80) | 3.7 (1.9, 5.8) |  | 10 (4, 15) | 4.0 (1.8, 6.3) |
|  |  |  | 65 | 33 (18, 53) | 2.9 (1.5, 4.6) |  | 107 (55, 166) | 3.6 (1.9, 5.7) |  | 19 (10, 31) | 3.5 (1.9, 5.7) |
|  |  |  | 75+ | 184 (112, 265) | 1.7 (1.0, 2.4) |  | 573 (359, 837) | 2.3 (1.5, 3.4) |  | 71 (41, 106) | 1.5 (0.9, 2.2) |
|  | Stroke, total | Males | 25 | 2 (1, 4) | 3.8 (2.1, 6.1) |  | 9 (5, 14) | 4.4 (2.5, 6.7) |  | 1 (1, 2) | 4.1 (2.4, 6.3) |
|  |  |  | 35 | 7 (4, 11) | 3.1 (1.8, 4.8) |  | 20 (12, 28) | 3.2 (2.0, 4.6) |  | 6 (3, 9) | 5.6 (3.0, 8.3) |
|  |  |  | 45 | 21 (13, 31) | 2.9 (1.8, 4.2) |  | 89 (57, 130) | 4.6 (2.9, 6.7) |  | 14 (8, 19) | 4.2 (2.5, 5.8) |
|  |  |  | 55 | 51 (34, 71) | 3.9 (2.6, 5.4) |  | 128 (81, 177) | 3.2 (2.0, 4.5) |  | 35 (23, 48) | 3.8 (2.4, 5.2) |
|  |  |  | 65 | 50 (31, 74) | 2.3 (1.4, 3.4) |  | 137 (91, 193) | 2.5 (1.7, 3.6) |  | 68 (45, 92) | 3.9 (2.6, 5.4) |
|  |  |  | 75+ | 114 (78, 157) | 1.4 (0.9, 1.9) |  | 340 (238, 455) | 2.1 (1.5, 2.8) |  | 119 (81, 158) | 1.7 (1.2, 2.3) |
|  |  | Females | 25 | 1 (1, 2) | 2.7 (1.4, 4.2) |  | 6 (4, 9) | 4.1 (2.6, 6.2) |  | 2 (1, 3) | 5.2 (3.4, 7.1) |
|  |  |  | 35 | 7 (4, 11) | 4.5 (2.6, 7.0) |  | 16 (10, 23) | 3.4 (2.1, 4.9) |  | 8 (4, 11) | 6.0 (3.4, 9.1) |
|  |  |  | 45 | 19 (12, 28) | 4.0 (2.5, 5.8) |  | 73 (47, 103) | 4.2 (2.7, 6.0) |  | 13 (8, 19) | 4.5 (2.6, 6.4) |
|  |  |  | 55 | 36 (23, 51) | 4.1 (2.6, 5.9) |  | 112 (75, 156) | 3.6 (2.4, 5.1) |  | 26 (16, 37) | 4.0 (2.4, 5.7) |
|  |  |  | 65 | 56 (37, 79) | 2.9 (1.9, 4.1) |  | 198 (135, 275) | 3.7 (2.5, 5.1) |  | 39 (25, 56) | 3.6 (2.3, 5.1) |
|  |  |  | 75+ | 262 (180, 354) | 1.7 (1.2, 2.3) |  | 841 (590, 1127) | 2.4 (1.7, 3.2) |  | 104 (70, 142) | 1.5 (1.0, 2.0) |
|  | CMD, total | Males | 25 | 4 (2, 6) | 0.8 (0.5, 1.1) |  | 14 (9, 20) | 0.9 (0.6, 1.2) |  | 2 (1, 3) | 0.8 (0.5, 1.1) |
|  |  |  | 35 | 13 (8, 18) | 0.8 (0.5, 1.1) |  | 39 (26, 54) | 0.7 (0.5, 0.9) |  | 11 (7, 16) | 1.2 (0.8, 1.7) |
|  |  |  | 45 | 44 (28, 63) | 0.8 (0.5, 1.1) |  | 201 (126, 297) | 1.0 (0.7, 1.5) |  | 31 (19, 45) | 1.0 (0.6, 1.4) |
|  |  |  | 55 | 106 (67, 150) | 1.0 (0.7, 1.5) |  | 296 (182, 433) | 0.8 (0.5, 1.2) |  | 83 (51, 117) | 0.9 (0.6, 1.3) |
|  |  |  | 65 | 101 (64, 147) | 0.7 (0.4, 1.0) |  | 289 (189, 417) | 0.7 (0.5, 1.0) |  | 130 (87, 181) | 1.1 (0.7, 1.5) |
|  |  |  | 75+ | 216 (150, 291) | 0.5 (0.3, 0.6) |  | 638 (439, 865) | 0.6 (0.4, 0.9) |  | 212 (147, 280) | 0.5 (0.4, 0.7) |
|  |  | Females | 25 | 2 (1, 2) | 0.6 (0.4, 0.9) |  | 8 (5, 11) | 1.0 (0.7, 1.4) |  | 2 (2, 3) | 1.4 (1.0, 1.9) |
|  |  |  | 35 | 10 (6, 14) | 1.2 (0.8, 1.8) |  | 24 (15, 33) | 0.9 (0.6, 1.2) |  | 9 (6, 13) | 1.9 (1.2, 2.8) |
|  |  |  | 45 | 31 (20, 42) | 1.2 (0.8, 1.6) |  | 109 (73, 147) | 1.2 (0.8, 1.6) |  | 18 (11, 25) | 1.3 (0.8, 1.8) |
|  |  |  | 55 | 59 (39, 84) | 1.1 (0.8, 1.6) |  | 186 (126, 254) | 1.0 (0.7, 1.4) |  | 40 (26, 54) | 1.1 (0.7, 1.5) |
|  |  |  | 65 | 91 (62, 126) | 0.9 (0.6, 1.3) |  | 313 (215, 415) | 1.1 (0.8, 1.5) |  | 57 (39, 77) | 1.1 (0.8, 1.5) |
|  |  |  | 75+ | 418 (303, 553) | 0.6 (0.4, 0.8) |  | 1278 (921, 1667) | 0.8 (0.6, 1.1) |  | 153 (106, 201) | 0.6 (0.4, 0.7) |
|  |  |  |  |  |  |  |  |  |  |  |  |
| **Vegetables** | CHD | Males | 25 | 3 (1, 4) | 1.5 (0.6, 2.6) |  | 8 (3, 13) | 1.5 (0.6, 2.5) |  | 1 (1, 2) | 1.8 (0.8, 2.9) |
|  |  |  | 35 | 14 (6, 23) | 1.9 (0.8, 3.2) |  | 37 (16, 63) | 1.4 (0.6, 2.4) |  | 7 (3, 12) | 1.8 (0.8, 2.9) |
|  |  |  | 45 | 44 (17, 76) | 1.4 (0.5, 2.4) |  | 140 (51, 234) | 1.3 (0.5, 2.1) |  | 33 (12, 56) | 1.8 (0.7, 3.0) |
|  |  |  | 55 | 65 (28, 106) | 1.1 (0.5, 1.8) |  | 239 (97, 399) | 1.1 (0.4, 1.8) |  | 67 (28, 112) | 1.2 (0.5, 2.1) |
|  |  |  | 65 | 82 (36, 139) | 0.9 (0.4, 1.6) |  | 253 (109, 431) | 1.0 (0.4, 1.8) |  | 82 (33, 138) | 1.2 (0.5, 2.0) |
|  |  |  | 75+ | 165 (87, 265) | 0.6 (0.3, 1.0) |  | 302 (152, 484) | 0.5 (0.3, 0.8) |  | 111 (58, 180) | 0.5 (0.3, 0.8) |
|  |  | Females | 25 | 1 (0, 2) | 1.7 (0.7, 2.9) |  | 3 (1, 4) | 1.6 (0.7, 2.6) |  | 1 (0, 1) | 1.7 (0.7, 2.8) |
|  |  |  | 35 | 5 (2, 8) | 1.6 (0.7, 2.7) |  | 15 (7, 24) | 1.6 (0.8, 2.7) |  | 2 (1, 3) | 1.8 (0.8, 2.8) |
|  |  |  | 45 | 18 (8, 30) | 1.5 (0.7, 2.6) |  | 58 (24, 99) | 1.6 (0.6, 2.6) |  | 10 (4, 17) | 2.1 (0.8, 3.4) |
|  |  |  | 55 | 31 (12, 52) | 1.3 (0.5, 2.1) |  | 112 (47, 188) | 1.3 (0.5, 2.1) |  | 20 (9, 32) | 1.3 (0.5, 2.0) |
|  |  |  | 65 | 56 (23, 96) | 1.2 (0.5, 2.0) |  | 141 (65, 233) | 1.0 (0.5, 1.7) |  | 27 (12, 45) | 1.2 (0.5, 2.0) |
|  |  |  | 75+ | 190 (101, 314) | 0.6 (0.3, 0.9) |  | 425 (214, 666) | 0.6 (0.3, 0.9) |  | 66 (34, 113) | 0.5 (0.3, 0.9) |
|  | Ischemic | Males | 25 | 0 (0, 0) | 5.5 (2.0, 9.3) |  | 1 (1, 2) | 5.4 (2.2, 8.9) |  | 0 (0, 0) | 6.5 (2.6, 10.7) |
|  | stroke |  | 35 | 1 (0, 2) | 6.6 (2.3, 11.2) |  | 3 (1, 5) | 5.1 (2.0, 8.3) |  | 0 (0, 1) | 6.2 (2.7, 10.2) |
|  |  |  | 45 | 4 (1, 6) | 5.1 (1.9, 8.2) |  | 10 (4, 17) | 4.4 (1.6, 7.2) |  | 3 (1, 4) | 6.5 (2.6, 11.3) |
|  |  |  | 55 | 6 (3, 10) | 3.9 (1.6, 6.4) |  | 17 (8, 29) | 3.9 (1.7, 6.5) |  | 5 (2, 8) | 4.4 (1.8, 7.4) |
|  |  |  | 65 | 9 (4, 15) | 3.3 (1.4, 5.4) |  | 23 (10, 39) | 3.9 (1.6, 6.6) |  | 8 (3, 14) | 4.3 (1.7, 7.2) |
|  |  |  | 75+ | 24 (11, 40) | 2.2 (1.0, 3.6) |  | 40 (18, 65) | 1.9 (0.9, 3.2) |  | 17 (8, 28) | 1.9 (0.9, 3.2) |
|  |  | Females | 25 | 0 (0, 1) | 5.8 (2.4, 10.2) |  | 1 (0, 1) | 5.3 (2.1, 8.6) |  | 1 (0, 1) | 6.1 (2.6, 9.6) |
|  |  |  | 35 | 0 (0, 1) | 5.8 (2.4, 9.5) |  | 3 (1, 5) | 5.7 (2.3, 9.4) |  | 1 (0, 2) | 6.1 (2.4, 9.9) |
|  |  |  | 45 | 2 (1, 4) | 5.2 (2.2, 8.6) |  | 9 (4, 15) | 5.7 (2.5, 9.6) |  | 2 (1, 3) | 7.6 (3.1, 12.4) |
|  |  |  | 55 | 5 (2, 8) | 4.3 (1.8, 7.5) |  | 13 (5, 21) | 4.6 (1.9, 7.4) |  | 3 (1, 4) | 4.5 (1.6, 7.4) |
|  |  |  | 65 | 11 (4, 18) | 4.2 (1.7, 7.1) |  | 23 (9, 37) | 3.8 (1.5, 6.2) |  | 4 (2, 7) | 4.3 (1.7, 7.2) |
|  |  |  | 75+ | 43 (20, 70) | 2.0 (0.9, 3.3) |  | 100 (47, 160) | 2.1 (1.0, 3.4) |  | 19 (9, 31) | 2.0 (0.9, 3.3) |
|  | Hemorrhagic | Males | 25 | 3 (0, 5) | 5.4 (0.8, 9.9) |  | 8 (2, 14) | 5.4 (1.2, 9.6) |  | 2 (0, 3) | 6.6 (1.8, 11.9) |
|  | stroke |  | 35 | 11 (2, 19) | 6.7 (1.3, 12.2) |  | 20 (5, 35) | 5.1 (1.2, 9.2) |  | 5 (1, 10) | 6.2 (1.3, 11.2) |
|  |  |  | 45 | 21 (3, 38) | 5.2 (0.8, 9.2) |  | 49 (12, 86) | 4.6 (1.1, 8.0) |  | 13 (3, 24) | 6.5 (1.8, 12.2) |
|  |  |  | 55 | 19 (4, 34) | 3.7 (0.8, 6.9) |  | 63 (13, 108) | 3.9 (0.8, 6.7) |  | 18 (3, 32) | 4.4 (0.7, 7.9) |
|  |  |  | 65 | 18 (4, 35) | 3.3 (0.8, 6.3) |  | 63 (18, 125) | 3.8 (1.1, 7.5) |  | 27 (6, 50) | 4.2 (0.9, 7.9) |
|  |  |  | 75+ | 33 (11, 57) | 2.2 (0.7, 3.9) |  | 70 (21, 126) | 1.9 (0.6, 3.5) |  | 31 (10, 58) | 1.9 (0.6, 3.6) |
|  |  | Females | 25 | 2 (0, 4) | 5.8 (1.0, 10.5) |  | 6 (1, 10) | 5.5 (0.8, 9.6) |  | 1 (0, 2) | 6.0 (1.2, 10.7) |
|  |  |  | 35 | 6 (1, 11) | 5.8 (1.3, 10.9) |  | 18 (4, 32) | 5.7 (1.1, 10.1) |  | 5 (1, 9) | 6.1 (1.2, 10.8) |
|  |  |  | 45 | 15 (3, 26) | 5.4 (1.2, 9.6) |  | 62 (13, 113) | 5.7 (1.2, 10.4) |  | 15 (3, 27) | 7.7 (1.6, 13.7) |
|  |  |  | 55 | 15 (3, 28) | 4.4 (1.0, 8.2) |  | 66 (17, 115) | 4.6 (1.2, 8.1) |  | 15 (4, 27) | 4.4 (1.1, 7.9) |
|  |  |  | 65 | 22 (5, 42) | 4.2 (0.9, 7.9) |  | 72 (13, 128) | 3.9 (0.7, 6.9) |  | 19 (4, 35) | 4.3 (0.9, 7.9) |
|  |  |  | 75+ | 50 (16, 91) | 2.0 (0.7, 3.7) |  | 133 (38, 240) | 2.1 (0.6, 3.7) |  | 27 (8, 49) | 2.0 (0.6, 3.6) |
|  | Other stroke | Males | 25 | 1 (0, 1) | 5.4 (1.4, 9.8) |  | 2 (1, 3) | 5.4 (1.8, 9.3) |  | 0 (0, 1) | 6.4 (2.2, 11.2) |
|  |  |  | 35 | 3 (1, 5) | 7.0 (2.4, 11.7) |  | 8 (3, 15) | 5.1 (1.6, 8.9) |  | 1 (0, 2) | 6.2 (1.8, 10.7) |
|  |  |  | 45 | 13 (3, 25) | 5.1 (1.3, 9.4) |  | 28 (9, 49) | 4.4 (1.5, 7.7) |  | 6 (2, 11) | 6.5 (2.4, 11.3) |
|  |  |  | 55 | 25 (11, 43) | 3.8 (1.6, 6.4) |  | 73 (26, 120) | 3.9 (1.4, 6.4) |  | 18 (7, 31) | 4.4 (1.6, 7.4) |
|  |  |  | 65 | 46 (20, 77) | 3.3 (1.5, 5.6) |  | 123 (44, 207) | 3.9 (1.4, 6.6) |  | 38 (14, 64) | 4.3 (1.6, 7.2) |
|  |  |  | 75+ | 125 (65, 194) | 2.2 (1.1, 3.4) |  | 209 (100, 333) | 2.0 (0.9, 3.1) |  | 86 (46, 137) | 1.9 (1.0, 3.1) |
|  |  | Females | 25 | 0 (0, 1) | 5.7 (1.9, 10.4) |  | 2 (1, 3) | 5.3 (1.7, 8.8) |  | 0 (0, 1) | 6.1 (2.8, 9.5) |
|  |  |  | 35 | 3 (1, 5) | 5.9 (1.7, 10.4) |  | 6 (2, 11) | 5.8 (2.1, 10.1) |  | 1 (1, 2) | 6.2 (2.4, 10.3) |
|  |  |  | 45 | 9 (3, 16) | 5.3 (1.8, 9.2) |  | 27 (9, 50) | 5.6 (1.8, 10.4) |  | 5 (2, 9) | 7.5 (2.4, 13) |
|  |  |  | 55 | 18 (7, 30) | 4.3 (1.7, 7.2) |  | 63 (26, 109) | 4.6 (1.9, 7.9) |  | 11 (3, 18) | 4.5 (1.4, 7.7) |
|  |  |  | 65 | 50 (20, 80) | 4.3 (1.8, 6.9) |  | 108 (44, 185) | 3.7 (1.5, 6.3) |  | 24 (8, 41) | 4.4 (1.4, 7.4) |
|  |  |  | 75+ | 225 (119, 333) | 2.1 (1.1, 3.1) |  | 507 (285, 782) | 2.1 (1.2, 3.2) |  | 95 (49, 156) | 2.0 (1.0, 3.3) |
|  | Stroke, total | Males | 25 | 3 (1, 6) | 5.5 (1.9, 8.9) |  | 11 (5, 17) | 5.4 (2.3, 8.6) |  | 2 (1, 3) | 6.5 (3, 10.4) |
|  |  |  | 35 | 15 (6, 24) | 6.7 (2.8, 10.9) |  | 31 (15, 47) | 5.1 (2.4, 7.7) |  | 7 (3, 11) | 6.2 (2.6, 10) |
|  |  |  | 45 | 38 (18, 59) | 5.1 (2.4, 7.9) |  | 87 (44, 131) | 4.5 (2.3, 6.7) |  | 22 (11, 33) | 6.6 (3.3, 10.1) |
|  |  |  | 55 | 50 (29, 74) | 3.8 (2.2, 5.7) |  | 153 (84, 221) | 3.9 (2.1, 5.6) |  | 41 (23, 61) | 4.4 (2.5, 6.5) |
|  |  |  | 65 | 74 (45, 109) | 3.4 (2.0, 4.9) |  | 211 (114, 321) | 3.9 (2.1, 5.9) |  | 74 (40, 107) | 4.3 (2.3, 6.2) |
|  |  |  | 75+ | 182 (117, 255) | 2.2 (1.4, 3.1) |  | 317 (203, 457) | 1.9 (1.2, 2.8) |  | 134 (87, 193) | 1.9 (1.3, 2.8) |
|  |  | Females | 25 | 3 (1, 4) | 5.8 (2.2, 9.4) |  | 8 (3, 13) | 5.5 (2.2, 8.4) |  | 2 (1, 3) | 6.1 (3.5, 8.7) |
|  |  |  | 35 | 9 (4, 15) | 5.8 (2.6, 9.6) |  | 27 (12, 43) | 5.7 (2.5, 9.0) |  | 8 (4, 12) | 6.1 (2.9, 9.4) |
|  |  |  | 45 | 26 (14, 40) | 5.4 (2.9, 8.2) |  | 100 (47, 154) | 5.8 (2.7, 8.9) |  | 22 (10, 34) | 7.6 (3.3, 11.8) |
|  |  |  | 55 | 39 (23, 55) | 4.4 (2.6, 6.3) |  | 141 (77, 210) | 4.6 (2.5, 6.8) |  | 28 (14, 43) | 4.5 (2.2, 6.7) |
|  |  |  | 65 | 83 (48, 119) | 4.3 (2.5, 6.2) |  | 205 (113, 304) | 3.8 (2.1, 5.6) |  | 48 (26, 70) | 4.4 (2.3, 6.4) |
|  |  |  | 75+ | 320 (206, 435) | 2.1 (1.3, 2.8) |  | 747 (475, 1042) | 2.1 (1.3, 2.9) |  | 142 (89, 204) | 2.0 (1.3, 2.9) |
|  | CMD, total | Males | 25 | 6 (3, 9) | 1.1 (0.7, 1.7) |  | 19 (11, 27) | 1.2 (0.7, 1.7) |  | 3 (2, 5) | 1.3 (0.8, 1.9) |
|  |  |  | 35 | 29 (17, 42) | 1.7 (1.0, 2.5) |  | 68 (42, 100) | 1.2 (0.7, 1.7) |  | 15 (9, 21) | 1.6 (0.9, 2.2) |
|  |  |  | 45 | 82 (50, 122) | 1.4 (0.9, 2.1) |  | 228 (136, 331) | 1.2 (0.7, 1.7) |  | 56 (32, 82) | 1.7 (1.0, 2.5) |
|  |  |  | 55 | 116 (72, 161) | 1.1 (0.7, 1.6) |  | 398 (225, 561) | 1.1 (0.6, 1.5) |  | 109 (63, 157) | 1.2 (0.7, 1.7) |
|  |  |  | 65 | 156 (101, 222) | 1.0 (0.7, 1.4) |  | 466 (292, 660) | 1.1 (0.7, 1.6) |  | 156 (99, 223) | 1.3 (0.8, 1.8) |
|  |  |  | 75+ | 346 (247, 470) | 0.7 (0.5, 1.0) |  | 624 (431, 839) | 0.6 (0.4, 0.8) |  | 249 (169, 335) | 0.6 (0.4, 0.9) |
|  |  | Females | 25 | 4 (2, 5) | 1.4 (0.8, 2.1) |  | 11 (6, 16) | 1.3 (0.7, 1.9) |  | 3 (2, 4) | 1.7 (1.1, 2.3) |
|  |  |  | 35 | 14 (8, 20) | 1.7 (1.0, 2.5) |  | 42 (24, 60) | 1.6 (0.9, 2.2) |  | 10 (6, 14) | 2.0 (1.2, 3.0) |
|  |  |  | 45 | 44 (28, 62) | 1.7 (1.1, 2.4) |  | 156 (96, 224) | 1.7 (1.1, 2.5) |  | 33 (18, 47) | 2.4 (1.3, 3.5) |
|  |  |  | 55 | 70 (44, 95) | 1.4 (0.9, 1.9) |  | 251 (160, 356) | 1.4 (0.9, 1.9) |  | 49 (31, 67) | 1.4 (0.9, 1.9) |
|  |  |  | 65 | 140 (90, 189) | 1.4 (0.9, 1.9) |  | 345 (227, 481) | 1.2 (0.8, 1.7) |  | 74 (47, 103) | 1.5 (0.9, 2.0) |
|  |  |  | 75+ | 508 (364, 677) | 0.7 (0.5, 1.0) |  | 1179 (823, 1552) | 0.8 (0.5, 1.0) |  | 211 (141, 290) | 0.8 (0.5, 1.1) |
|  |  |  |  |  |  |  |  |  |  |  |  |
| **Nuts/seeds** | CHD | Males | 25 | 2 (0, 3) | 0.9 (0.2, 1.8) |  | 8 (4, 15) | 1.6 (0.8, 2.8) |  | 1 (1, 2) | 1.9 (0.9, 3.0) |
|  |  |  | 35 | 12 (5, 20) | 1.6 (0.7, 2.8) |  | 57 (33, 84) | 2.2 (1.2, 3.2) |  | 6 (3, 10) | 1.5 (0.8, 2.4) |
|  |  |  | 45 | 20 (6, 43) | 0.6 (0.2, 1.4) |  | 123 (64, 204) | 1.1 (0.6, 1.9) |  | 20 (9, 32) | 1.1 (0.5, 1.7) |
|  |  |  | 55 | 46 (19, 82) | 0.8 (0.3, 1.4) |  | 317 (155, 503) | 1.4 (0.7, 2.3) |  | 59 (34, 89) | 1.1 (0.6, 1.6) |
|  |  |  | 65 | 37 (17, 69) | 0.4 (0.2, 0.8) |  | 253 (141, 387) | 1.0 (0.6, 1.6) |  | 64 (35, 94) | 0.9 (0.5, 1.3) |
|  |  |  | 75+ | 182 (88, 301) | 0.7 (0.3, 1.1) |  | 534 (332, 783) | 0.9 (0.6, 1.4) |  | 114 (52, 175) | 0.5 (0.2, 0.8) |
|  |  | Females | 25 | 0 (0, 1) | 0.8 (0.4, 1.3) |  | 2 (1, 3) | 1.1 (0.6, 1.9) |  | 1 (0, 1) | 1.5 (0.7, 2.6) |
|  |  |  | 35 | 6 (2, 11) | 2.0 (0.8, 3.7) |  | 5 (1, 11) | 0.5 (0.1, 1.2) |  | 2 (1, 3) | 1.8 (1.0, 2.8) |
|  |  |  | 45 | 11 (4, 21) | 1.0 (0.3, 1.8) |  | 106 (62, 158) | 2.8 (1.7, 4.2) |  | 7 (3, 10) | 1.3 (0.7, 2.1) |
|  |  |  | 55 | 24 (7, 45) | 1.0 (0.3, 1.9) |  | 147 (85, 213) | 1.7 (1.0, 2.4) |  | 24 (14, 35) | 1.5 (0.9, 2.2) |
|  |  |  | 65 | 29 (12, 55) | 0.6 (0.3, 1.1) |  | 242 (148, 357) | 1.8 (1.1, 2.6) |  | 12 (1, 19) | 0.5 (0.1, 0.8) |
|  |  |  | 75+ | 35 (2, 94) | 0.1 (0, 0.3) |  | 521 (319, 801) | 0.7 (0.4, 1.1) |  | 83 (50, 119) | 0.7 (0.4, 1.0) |
|  | Diabetes | Males | 25 | 0 (0, 1) | 0.4 (0.1, 0.9) |  | 2 (1, 3) | 0.7 (0.3, 1.4) |  | 0 (0, 0) | 0.8 (0.3, 1.4) |
|  |  |  | 35 | 2 (1, 3) | 0.7 (0.2, 1.3) |  | 7 (3, 12) | 0.9 (0.4, 1.6) |  | 1 (0, 1) | 0.7 (0.3, 1.1) |
|  |  |  | 45 | 2 (1, 4) | 0.3 (0.1, 0.6) |  | 11 (4, 20) | 0.5 (0.2, 0.9) |  | 2 (1, 3) | 0.5 (0.2, 0.9) |
|  |  |  | 55 | 4 (2, 8) | 0.3 (0.1, 0.6) |  | 28 (12, 48) | 0.6 (0.3, 1.0) |  | 4 (2, 8) | 0.4 (0.2, 0.8) |
|  |  |  | 65 | 4 (1, 7) | 0.2 (0.1, 0.3) |  | 22 (10, 38) | 0.4 (0.2, 0.7) |  | 5 (2, 8) | 0.4 (0.2, 0.6) |
|  |  |  | 75+ | 12 (5, 21) | 0.3 (0.1, 0.5) |  | 29 (13, 48) | 0.4 (0.2, 0.6) |  | 5 (2, 9) | 0.2 (0.1, 0.4) |
|  |  | Females | 25 | 0 (0, 0) | 0.3 (0.1, 0.6) |  | 1 (0, 2) | 0.5 (0.2, 0.9) |  | 0 (0, 0) | 0.7 (0.3, 1.2) |
|  |  |  | 35 | 1 (0, 2) | 0.9 (0.3, 1.8) |  | 1 (0, 3) | 0.2 (0, 0.6) |  | 0 (0, 1) | 0.8 (0.3, 1.3) |
|  |  |  | 45 | 2 (1, 4) | 0.4 (0.1, 0.9) |  | 17 (7, 28) | 1.2 (0.5, 2.0) |  | 1 (0, 2) | 0.6 (0.2, 1.0) |
|  |  |  | 55 | 4 (1, 9) | 0.4 (0.1, 0.9) |  | 21 (9, 35) | 0.7 (0.3, 1.2) |  | 3 (2, 5) | 0.6 (0.3, 1.0) |
|  |  |  | 65 | 4 (2, 8) | 0.3 (0.1, 0.5) |  | 30 (14, 50) | 0.7 (0.3, 1.2) |  | 1 (0, 3) | 0.2 (0, 0.4) |
|  |  |  | 75+ | 2 (0, 8) | 0 (0, 0.1) |  | 31 (16, 48) | 0.3 (0.2, 0.5) |  | 4 (2, 7) | 0.3 (0.1, 0.4) |
|  | CMD, total | Males | 25 | 2 (1, 3) | 0.3 (0.1, 0.7) |  | 10 (5, 16) | 0.6 (0.3, 1.0) |  | 2 (1, 3) | 0.6 (0.3, 1.0) |
|  |  |  | 35 | 14 (6, 22) | 0.8 (0.4, 1.3) |  | 63 (39, 93) | 1.1 (0.7, 1.6) |  | 7 (4, 11) | 0.7 (0.4, 1.1) |
|  |  |  | 45 | 22 (8, 46) | 0.4 (0.1, 0.8) |  | 133 (75, 212) | 0.7 (0.4, 1.1) |  | 21 (11, 34) | 0.6 (0.3, 1.0) |
|  |  |  | 55 | 50 (24, 86) | 0.5 (0.2, 0.8) |  | 346 (186, 535) | 0.9 (0.5, 1.4) |  | 64 (38, 95) | 0.7 (0.4, 1.0) |
|  |  |  | 65 | 42 (21, 73) | 0.3 (0.1, 0.5) |  | 274 (162, 410) | 0.7 (0.4, 1.0) |  | 69 (40, 99) | 0.6 (0.3, 0.8) |
|  |  |  | 75+ | 193 (99, 311) | 0.4 (0.2, 0.7) |  | 563 (356, 812) | 0.6 (0.4, 0.8) |  | 120 (57, 180) | 0.3 (0.1, 0.5) |
|  |  | Females | 25 | 1 (0, 1) | 0.3 (0.2, 0.4) |  | 3 (2, 4) | 0.4 (0.2, 0.5) |  | 1 (0, 1) | 0.4 (0.2, 0.6) |
|  |  |  | 35 | 7 (4, 12) | 0.9 (0.4, 1.5) |  | 6 (1, 12) | 0.2 (0, 0.4) |  | 3 (2, 4) | 0.5 (0.3, 0.8) |
|  |  |  | 45 | 13 (6, 23) | 0.5 (0.2, 0.9) |  | 122 (78, 173) | 1.3 (0.9, 1.9) |  | 8 (4, 11) | 0.6 (0.3, 0.8) |
|  |  |  | 55 | 29 (11, 50) | 0.6 (0.2, 1.0) |  | 167 (104, 237) | 0.9 (0.6, 1.3) |  | 28 (17, 39) | 0.8 (0.5, 1.1) |
|  |  |  | 65 | 34 (16, 60) | 0.3 (0.2, 0.6) |  | 273 (176, 389) | 1.0 (0.6, 1.4) |  | 13 (3, 21) | 0.3 (0.1, 0.4) |
|  |  |  | 75+ | 38 (5, 96) | 0.1 (0, 0.1) |  | 554 (350, 833) | 0.4 (0.2, 0.5) |  | 87 (54, 125) | 0.3 (0.2, 0.5) |
|  |  |  |  |  |  |  |  |  |  |  |  |
| **Whole grains** | CHD | Males | 25 | 0 (0, 1) | 0.2 (0, 0.5) |  | 1 (0, 2) | 0.2 (0.1, 0.5) |  | 0 (0, 0) | 0.3 (0.1, 0.6) |
|  |  |  | 35 | 1 (0, 2) | 0.1 (0, 0.3) |  | 6 (1, 11) | 0.2 (0, 0.4) |  | 1 (0, 3) | 0.3 (0.1, 0.6) |
|  |  |  | 45 | 6 (1, 12) | 0.2 (0, 0.4) |  | 24 (4, 48) | 0.2 (0, 0.4) |  | 6 (1, 11) | 0.3 (0, 0.6) |
|  |  |  | 55 | 7 (1, 14) | 0.1 (0, 0.2) |  | 51 (8, 101) | 0.2 (0, 0.5) |  | 13 (2, 26) | 0.2 (0, 0.5) |
|  |  |  | 65 | 11 (2, 22) | 0.1 (0, 0.3) |  | 55 (10, 101) | 0.2 (0, 0.4) |  | 13 (3, 25) | 0.2 (0, 0.4) |
|  |  |  | 75+ | 30 (9, 57) | 0.1 (0, 0.2) |  | 81 (25, 151) | 0.1 (0, 0.3) |  | 24 (7, 45) | 0.1 (0, 0.2) |
|  |  | Females | 25 | 0 (0, 0) | 0.2 (0, 0.5) |  | 0 (0, 1) | 0.2 (0, 0.4) |  | 0 (0, 0) | 0.4 (0.1, 0.7) |
|  |  |  | 35 | 1 (0, 1) | 0.2 (0, 0.4) |  | 2 (1, 4) | 0.2 (0.1, 0.5) |  | 0 (0, 1) | 0.4 (0.1, 0.7) |
|  |  |  | 45 | 2 (0, 4) | 0.2 (0, 0.4) |  | 7 (1, 13) | 0.2 (0, 0.4) |  | 1 (0, 2) | 0.3 (0, 0.5) |
|  |  |  | 55 | 6 (1, 12) | 0.2 (0, 0.5) |  | 18 (4, 36) | 0.2 (0, 0.4) |  | 4 (1, 7) | 0.2 (0, 0.4) |
|  |  |  | 65 | 8 (2, 17) | 0.2 (0, 0.4) |  | 27 (4, 50) | 0.2 (0, 0.4) |  | 4 (1, 8) | 0.2 (0, 0.4) |
|  |  |  | 75+ | 47 (12, 87) | 0.1 (0, 0.3) |  | 99 (32, 177) | 0.1 (0, 0.2) |  | 18 (5, 33) | 0.1 (0, 0.3) |
|  | Ischemic | Males | 25 | 0 (0, 0) | 0.5 (0.1, 1.2) |  | 0 (0, 0) | 0.6 (0.2, 1.1) |  | 0 (0, 0) | 0.8 (0.3, 1.5) |
|  | stroke |  | 35 | 0 (0, 0) | 0.3 (0.1, 0.7) |  | 0 (0, 1) | 0.6 (0.2, 1.0) |  | 0 (0, 0) | 0.8 (0.3, 1.5) |
|  |  |  | 45 | 0 (0, 1) | 0.5 (0.2, 0.9) |  | 1 (0, 2) | 0.6 (0.2, 1.0) |  | 0 (0, 1) | 0.8 (0.3, 1.5) |
|  |  |  | 55 | 0 (0, 1) | 0.3 (0.1, 0.6) |  | 3 (1, 5) | 0.6 (0.2, 1.2) |  | 1 (0, 1) | 0.6 (0.2, 1.2) |
|  |  |  | 65 | 1 (0, 2) | 0.3 (0.1, 0.6) |  | 3 (1, 6) | 0.6 (0.2, 1.0) |  | 1 (0, 2) | 0.5 (0.2, 0.9) |
|  |  |  | 75+ | 3 (1, 6) | 0.3 (0.1, 0.5) |  | 7 (3, 13) | 0.4 (0.1, 0.6) |  | 3 (1, 5) | 0.3 (0.1, 0.5) |
|  |  | Females | 25 | 0 (0, 0) | 0.6 (0.2, 1.1) |  | 0 (0, 0) | 0.6 (0.1, 1.0) |  | 0 (0, 0) | 0.9 (0.3, 1.7) |
|  |  |  | 35 | 0 (0, 0) | 0.5 (0.2, 1.0) |  | 0 (0, 1) | 0.7 (0.2, 1.2) |  | 0 (0, 0) | 0.9 (0.3, 1.7) |
|  |  |  | 45 | 0 (0, 0) | 0.5 (0.1, 0.9) |  | 1 (0, 1) | 0.5 (0.2, 0.9) |  | 0 (0, 0) | 0.7 (0.2, 1.2) |
|  |  |  | 55 | 1 (0, 1) | 0.6 (0.2, 1.3) |  | 2 (1, 3) | 0.6 (0.2, 1.0) |  | 0 (0, 1) | 0.6 (0.2, 1.1) |
|  |  |  | 65 | 1 (0, 2) | 0.5 (0.1, 0.9) |  | 3 (1, 5) | 0.5 (0.2, 0.9) |  | 0 (0, 1) | 0.5 (0.2, 0.9) |
|  |  |  | 75+ | 8 (4, 14) | 0.4 (0.2, 0.6) |  | 17 (7, 28) | 0.3 (0.1, 0.6) |  | 4 (2, 7) | 0.4 (0.2, 0.7) |
|  | Hemorrhagic | Males | 25 | 0 (0, 1) | 0.5 (0.1, 1.2) |  | 1 (0, 2) | 0.6 (0.2, 1.1) |  | 0 (0, 0) | 0.9 (0.3, 1.6) |
|  | stroke |  | 35 | 1 (0, 1) | 0.3 (0.1, 0.6) |  | 2 (1, 4) | 0.6 (0.2, 1.1) |  | 1 (0, 1) | 0.9 (0.3, 1.5) |
|  |  |  | 45 | 2 (1, 4) | 0.5 (0.2, 0.8) |  | 6 (2, 11) | 0.6 (0.2, 1.0) |  | 2 (1, 3) | 0.8 (0.3, 1.5) |
|  |  |  | 55 | 2 (0, 3) | 0.3 (0.1, 0.6) |  | 10 (4, 18) | 0.6 (0.2, 1.1) |  | 3 (1, 5) | 0.7 (0.2, 1.2) |
|  |  |  | 65 | 2 (1, 3) | 0.3 (0.1, 0.6) |  | 9 (3, 17) | 0.6 (0.2, 1.0) |  | 3 (1, 6) | 0.5 (0.2, 0.9) |
|  |  |  | 75+ | 4 (2, 7) | 0.3 (0.1, 0.5) |  | 13 (6, 23) | 0.4 (0.2, 0.6) |  | 5 (2, 8) | 0.3 (0.1, 0.5) |
|  |  | Females | 25 | 0 (0, 0) | 0.6 (0.2, 1.1) |  | 1 (0, 1) | 0.6 (0.2, 1.0) |  | 0 (0, 0) | 1.0 (0.3, 1.7) |
|  |  |  | 35 | 1 (0, 1) | 0.5 (0.2, 1.0) |  | 2 (1, 4) | 0.7 (0.2, 1.2) |  | 1 (0, 1) | 0.9 (0.3, 1.7) |
|  |  |  | 45 | 1 (0, 2) | 0.5 (0.2, 0.9) |  | 5 (2, 10) | 0.5 (0.2, 0.9) |  | 1 (0, 2) | 0.7 (0.2, 1.3) |
|  |  |  | 55 | 2 (1, 4) | 0.6 (0.2, 1.2) |  | 8 (2, 14) | 0.6 (0.2, 1.0) |  | 2 (1, 4) | 0.6 (0.2, 1.1) |
|  |  |  | 65 | 2 (1, 5) | 0.5 (0.2, 0.9) |  | 9 (3, 17) | 0.5 (0.2, 0.9) |  | 2 (1, 4) | 0.5 (0.2, 1.0) |
|  |  |  | 75+ | 9 (4, 15) | 0.4 (0.2, 0.6) |  | 22 (9, 38) | 0.3 (0.1, 0.6) |  | 5 (2, 9) | 0.4 (0.1, 0.7) |
|  | Other stroke | Males | 25 | 0 (0, 0) | 0.5 (0.1, 1.2) |  | 0 (0, 0) | 0.6 (0.2, 1.0) |  | 0 (0, 0) | 0.9 (0.4, 1.5) |
|  |  |  | 35 | 0 (0, 0) | 0.4 (0.1, 0.6) |  | 1 (0, 2) | 0.6 (0.3, 1.0) |  | 0 (0, 0) | 0.9 (0.3, 1.5) |
|  |  |  | 45 | 1 (1, 2) | 0.5 (0.2, 0.9) |  | 4 (2, 6) | 0.6 (0.3, 1.0) |  | 1 (0, 1) | 0.8 (0.3, 1.5) |
|  |  |  | 55 | 2 (1, 4) | 0.3 (0.1, 0.6) |  | 11 (5, 20) | 0.6 (0.3, 1.1) |  | 3 (1, 5) | 0.6 (0.3, 1.1) |
|  |  |  | 65 | 5 (2, 8) | 0.3 (0.2, 0.5) |  | 18 (8, 29) | 0.6 (0.3, 0.9) |  | 4 (2, 8) | 0.5 (0.2, 0.9) |
|  |  |  | 75+ | 16 (9, 25) | 0.3 (0.2, 0.4) |  | 41 (20, 61) | 0.4 (0.2, 0.6) |  | 13 (7, 22) | 0.3 (0.2, 0.5) |
|  |  | Females | 25 | 0 (0, 0) | 0.6 (0.2, 1.0) |  | 0 (0, 0) | 0.6 (0.2, 1.0) |  | 0 (0, 0) | 0.9 (0.4, 1.5) |
|  |  |  | 35 | 0 (0, 0) | 0.5 (0.2, 1.0) |  | 1 (0, 1) | 0.7 (0.2, 1.1) |  | 0 (0, 0) | 0.9 (0.4, 1.5) |
|  |  |  | 45 | 1 (0, 1) | 0.5 (0.2, 0.9) |  | 2 (1, 4) | 0.5 (0.2, 0.8) |  | 0 (0, 1) | 0.7 (0.3, 1.2) |
|  |  |  | 55 | 3 (1, 5) | 0.6 (0.3, 1.1) |  | 8 (3, 13) | 0.6 (0.2, 1.0) |  | 1 (1, 2) | 0.6 (0.2, 1.0) |
|  |  |  | 65 | 5 (3, 9) | 0.5 (0.2, 0.8) |  | 15 (7, 25) | 0.5 (0.2, 0.9) |  | 3 (1, 5) | 0.5 (0.2, 0.9) |
|  |  |  | 75+ | 39 (21, 61) | 0.4 (0.2, 0.6) |  | 85 (47, 131) | 0.3 (0.2, 0.5) |  | 18 (10, 30) | 0.4 (0.2, 0.6) |
|  | Stroke, total | Males | 25 | 0 (0, 1) | 0.5 (0.2, 1.0) |  | 1 (1, 2) | 0.6 (0.3, 1.0) |  | 0 (0, 0) | 0.8 (0.4, 1.4) |
|  |  |  | 35 | 1 (0, 1) | 0.3 (0.2, 0.6) |  | 4 (2, 6) | 0.6 (0.3, 0.9) |  | 1 (0, 2) | 0.9 (0.4, 1.4) |
|  |  |  | 45 | 4 (2, 5) | 0.5 (0.3, 0.7) |  | 11 (7, 17) | 0.6 (0.3, 0.9) |  | 3 (1, 4) | 0.8 (0.4, 1.2) |
|  |  |  | 55 | 4 (2, 6) | 0.3 (0.2, 0.5) |  | 24 (15, 36) | 0.6 (0.4, 0.9) |  | 6 (3, 9) | 0.7 (0.4, 1.0) |
|  |  |  | 65 | 7 (4, 11) | 0.3 (0.2, 0.5) |  | 31 (19, 44) | 0.6 (0.3, 0.8) |  | 9 (5, 13) | 0.5 (0.3, 0.8) |
|  |  |  | 75+ | 24 (16, 33) | 0.3 (0.2, 0.4) |  | 62 (40, 86) | 0.4 (0.2, 0.5) |  | 21 (14, 30) | 0.3 (0.2, 0.4) |
|  |  | Females | 25 | 0 (0, 0) | 0.6 (0.2, 1.0) |  | 1 (0, 1) | 0.6 (0.3, 0.9) |  | 0 (0, 1) | 0.9 (0.6, 1.4) |
|  |  |  | 35 | 1 (0, 1) | 0.5 (0.3, 0.9) |  | 3 (2, 5) | 0.7 (0.3, 1.0) |  | 1 (1, 2) | 0.9 (0.5, 1.4) |
|  |  |  | 45 | 2 (1, 4) | 0.5 (0.3, 0.7) |  | 9 (5, 13) | 0.5 (0.3, 0.7) |  | 2 (1, 3) | 0.7 (0.3, 1.1) |
|  |  |  | 55 | 6 (3, 8) | 0.6 (0.4, 1.0) |  | 17 (10, 25) | 0.6 (0.3, 0.8) |  | 4 (2, 6) | 0.6 (0.3, 0.9) |
|  |  |  | 65 | 9 (6, 13) | 0.5 (0.3, 0.7) |  | 28 (17, 39) | 0.5 (0.3, 0.7) |  | 6 (3, 9) | 0.5 (0.3, 0.8) |
|  |  |  | 75+ | 57 (39, 81) | 0.4 (0.2, 0.5) |  | 125 (82, 173) | 0.4 (0.2, 0.5) |  | 28 (18, 40) | 0.4 (0.3, 0.6) |
|  | Diabetes | Males | 25 | 1 (0, 1) | 0.7 (0.1, 1.6) |  | 2 (1, 3) | 0.8 (0.4, 1.3) |  | 0 (0, 1) | 1.2 (0.6, 1.9) |
|  |  |  | 35 | 1 (1, 2) | 0.5 (0.2, 0.8) |  | 6 (3, 10) | 0.8 (0.4, 1.3) |  | 1 (1, 2) | 1.2 (0.6, 1.9) |
|  |  |  | 45 | 5 (3, 8) | 0.7 (0.4, 1.1) |  | 19 (11, 29) | 0.8 (0.5, 1.3) |  | 4 (2, 6) | 1.1 (0.6, 1.8) |
|  |  |  | 55 | 6 (3, 10) | 0.4 (0.2, 0.8) |  | 41 (22, 65) | 0.9 (0.5, 1.4) |  | 9 (5, 15) | 0.9 (0.5, 1.5) |
|  |  |  | 65 | 10 (5, 16) | 0.5 (0.2, 0.7) |  | 41 (22, 64) | 0.8 (0.4, 1.3) |  | 9 (5, 15) | 0.7 (0.4, 1.1) |
|  |  |  | 75+ | 17 (10, 27) | 0.4 (0.2, 0.6) |  | 41 (24, 63) | 0.5 (0.3, 0.8) |  | 10 (6, 17) | 0.4 (0.2, 0.7) |
|  |  | Females | 25 | 0 (0, 1) | 0.8 (0.4, 1.4) |  | 1 (1, 2) | 0.8 (0.4, 1.4) |  | 0 (0, 0) | 1.3 (0.7, 2.1) |
|  |  |  | 35 | 1 (1, 2) | 0.8 (0.4, 1.3) |  | 4 (2, 7) | 0.9 (0.5, 1.5) |  | 1 (0, 1) | 1.3 (0.7, 2.1) |
|  |  |  | 45 | 3 (1, 5) | 0.7 (0.3, 1.1) |  | 10 (5, 15) | 0.7 (0.4, 1.1) |  | 2 (1, 3) | 1.0 (0.5, 1.5) |
|  |  |  | 55 | 8 (4, 14) | 0.9 (0.4, 1.5) |  | 24 (12, 38) | 0.8 (0.4, 1.3) |  | 4 (2, 7) | 0.8 (0.5, 1.3) |
|  |  |  | 65 | 11 (6, 18) | 0.7 (0.3, 1.0) |  | 29 (16, 47) | 0.7 (0.4, 1.2) |  | 5 (3, 8) | 0.7 (0.4, 1.2) |
|  |  |  | 75+ | 32 (19, 48) | 0.5 (0.3, 0.8) |  | 51 (29, 78) | 0.5 (0.3, 0.8) |  | 9 (5, 14) | 0.6 (0.3, 0.9) |
|  | CMD, total | Males | 25 | 1 (1, 2) | 0.2 (0.1, 0.4) |  | 4 (3, 6) | 0.3 (0.2, 0.4) |  | 1 (1, 1) | 0.3 (0.2, 0.5) |
|  |  |  | 35 | 3 (2, 4) | 0.2 (0.1, 0.3) |  | 16 (10, 22) | 0.3 (0.2, 0.4) |  | 3 (2, 5) | 0.4 (0.2, 0.5) |
|  |  |  | 45 | 14 (8, 21) | 0.2 (0.1, 0.4) |  | 54 (33, 80) | 0.3 (0.2, 0.4) |  | 12 (7, 18) | 0.4 (0.2, 0.6) |
|  |  |  | 55 | 17 (10, 25) | 0.2 (0.1, 0.2) |  | 117 (68, 171) | 0.3 (0.2, 0.5) |  | 29 (17, 44) | 0.3 (0.2, 0.5) |
|  |  |  | 65 | 29 (18, 42) | 0.2 (0.1, 0.3) |  | 126 (77, 182) | 0.3 (0.2, 0.4) |  | 32 (19, 45) | 0.3 (0.2, 0.4) |
|  |  |  | 75+ | 72 (47, 101) | 0.2 (0.1, 0.2) |  | 185 (119, 261) | 0.2 (0.1, 0.3) |  | 56 (36, 79) | 0.1 (0.1, 0.2) |
|  |  | Females | 25 | 1 (1, 1) | 0.3 (0.2, 0.5) |  | 3 (2, 4) | 0.3 (0.2, 0.5) |  | 1 (1, 1) | 0.5 (0.3, 0.6) |
|  |  |  | 35 | 3 (2, 4) | 0.3 (0.2, 0.4) |  | 10 (7, 13) | 0.4 (0.2, 0.5) |  | 2 (2, 3) | 0.5 (0.3, 0.7) |
|  |  |  | 45 | 7 (5, 10) | 0.3 (0.2, 0.4) |  | 26 (17, 35) | 0.3 (0.2, 0.4) |  | 5 (3, 7) | 0.4 (0.2, 0.5) |
|  |  |  | 55 | 20 (12, 28) | 0.4 (0.2, 0.5) |  | 60 (40, 83) | 0.3 (0.2, 0.5) |  | 12 (8, 16) | 0.3 (0.2, 0.5) |
|  |  |  | 65 | 29 (19, 40) | 0.3 (0.2, 0.4) |  | 85 (57, 117) | 0.3 (0.2, 0.4) |  | 15 (11, 21) | 0.3 (0.2, 0.4) |
|  |  |  | 75+ | 138 (96, 189) | 0.2 (0.1, 0.3) |  | 275 (197, 366) | 0.2 (0.1, 0.2) |  | 55 (37, 74) | 0.2 (0.1, 0.3) |
|  |  |  |  |  |  |  |  |  |  |  |  |
| **Processed** | CHD | Males | 25 | 2 (1, 6) | 1.4 (0.3, 3.4) |  | 7 (1, 16) | 1.3 (0.3, 3.0) |  | 1 (0, 2) | 1.0 (0.2, 2.6) |
| **meats** |  |  | 35 | 6 (2, 15) | 0.9 (0.2, 2.0) |  | 37 (9, 85) | 1.4 (0.3, 3.2) |  | 4 (1, 11) | 1.1 (0.3, 2.6) |
|  |  |  | 45 | 37 (9, 93) | 1.2 (0.3, 2.9) |  | 118 (25, 265) | 1.1 (0.2, 2.4) |  | 14 (4, 32) | 0.8 (0.2, 1.7) |
|  |  |  | 55 | 48 (11, 106) | 0.8 (0.2, 1.8) |  | 177 (50, 369) | 0.8 (0.2, 1.7) |  | 32 (8, 73) | 0.6 (0.1, 1.3) |
|  |  |  | 65 | 68 (17, 143) | 0.8 (0.2, 1.6) |  | 189 (45, 472) | 0.8 (0.2, 1.9) |  | 35 (8, 79) | 0.5 (0.1, 1.1) |
|  |  |  | 75+ | 122 (36, 268) | 0.4 (0.1, 1.0) |  | 151 (46, 331) | 0.3 (0.1, 0.6) |  | 61 (18, 135) | 0.3 (0.1, 0.6) |
|  |  | Females | 25 | 1 (0, 1) | 0.9 (0.2, 2.0) |  | 1 (0, 3) | 0.8 (0.2, 1.8) |  | 0 (0, 0) | 0.7 (0.2, 1.4) |
|  |  |  | 35 | 3 (1, 6) | 0.9 (0.2, 2.1) |  | 9 (2, 19) | 0.9 (0.2, 2.1) |  | 1 (0, 2) | 0.6 (0.1, 1.4) |
|  |  |  | 45 | 7 (2, 15) | 0.6 (0.1, 1.3) |  | 33 (8, 90) | 0.9 (0.2, 2.4) |  | 3 (1, 7) | 0.5 (0.1, 1.4) |
|  |  |  | 55 | 16 (3, 36) | 0.6 (0.1, 1.5) |  | 47 (11, 102) | 0.5 (0.1, 1.2) |  | 5 (1, 13) | 0.3 (0.1, 0.8) |
|  |  |  | 65 | 23 (6, 51) | 0.5 (0.1, 1.1) |  | 65 (15, 152) | 0.5 (0.1, 1.1) |  | 9 (2, 22) | 0.4 (0.1, 1.0) |
|  |  |  | 75+ | 86 (24, 187) | 0.3 (0.1, 0.5) |  | 165 (49, 347) | 0.2 (0.1, 0.5) |  | 18 (5, 42) | 0.1 (0, 0.3) |
|  | Diabetes | Males | 25 | 2 (1, 4) | 2.0 (0.7, 4.7) |  | 4 (1, 8) | 1.8 (0.6, 3.6) |  | 0 (0, 1) | 1.4 (0.4, 3.0) |
|  |  |  | 35 | 3 (1, 6) | 1.1 (0.4, 2.4) |  | 14 (4, 28) | 1.9 (0.5, 3.8) |  | 1 (0, 2) | 1.4 (0.5, 2.8) |
|  |  |  | 45 | 12 (4, 24) | 1.7 (0.5, 3.5) |  | 33 (11, 65) | 1.5 (0.5, 2.9) |  | 3 (1, 7) | 1.0 (0.3, 2.1) |
|  |  |  | 55 | 15 (5, 29) | 1.1 (0.4, 2.2) |  | 50 (17, 101) | 1.1 (0.4, 2.2) |  | 8 (2, 16) | 0.8 (0.2, 1.6) |
|  |  |  | 65 | 22 (8, 42) | 1.0 (0.4, 2.0) |  | 56 (18, 122) | 1.1 (0.3, 2.4) |  | 8 (3, 17) | 0.6 (0.2, 1.3) |
|  |  |  | 75+ | 26 (9, 51) | 0.6 (0.2, 1.2) |  | 28 (11, 55) | 0.4 (0.1, 0.7) |  | 9 (3, 18) | 0.4 (0.1, 0.7) |
|  |  | Females | 25 | 1 (0, 1) | 1.2 (0.4, 2.5) |  | 2 (1, 4) | 1.1 (0.4, 2.2) |  | 0 (0, 0) | 0.8 (0.3, 1.7) |
|  |  |  | 35 | 2 (1, 3) | 1.2 (0.4, 2.5) |  | 6 (2, 12) | 1.2 (0.4, 2.7) |  | 0 (0, 1) | 0.8 (0.3, 1.7) |
|  |  |  | 45 | 3 (1, 7) | 0.8 (0.2, 1.6) |  | 18 (6, 39) | 1.3 (0.4, 2.8) |  | 1 (0, 3) | 0.7 (0.2, 1.5) |
|  |  |  | 55 | 8 (3, 17) | 0.9 (0.3, 1.9) |  | 21 (7, 43) | 0.7 (0.2, 1.4) |  | 2 (1, 5) | 0.4 (0.1, 1.0) |
|  |  |  | 65 | 11 (4, 23) | 0.6 (0.2, 1.3) |  | 27 (10, 53) | 0.7 (0.2, 1.3) |  | 4 (1, 8) | 0.5 (0.2, 1.2) |
|  |  |  | 75+ | 21 (7, 43) | 0.3 (0.1, 0.7) |  | 31 (11, 60) | 0.3 (0.1, 0.6) |  | 3 (1, 6) | 0.2 (0.1, 0.4) |
|  | CMD, total | Males | 25 | 4 (2, 8) | 0.8 (0.3, 1.5) |  | 11 (5, 21) | 0.7 (0.3, 1.3) |  | 1 (1, 3) | 0.5 (0.2, 1.0) |
|  |  |  | 35 | 9 (4, 18) | 0.6 (0.2, 1.1) |  | 51 (21, 100) | 0.9 (0.4, 1.7) |  | 6 (2, 12) | 0.6 (0.2, 1.3) |
|  |  |  | 45 | 49 (19, 106) | 0.9 (0.3, 1.8) |  | 152 (57, 295) | 0.8 (0.3, 1.5) |  | 18 (7, 35) | 0.6 (0.2, 1.1) |
|  |  |  | 55 | 63 (24, 122) | 0.6 (0.2, 1.2) |  | 233 (92, 425) | 0.6 (0.2, 1.1) |  | 41 (15, 83) | 0.5 (0.2, 0.9) |
|  |  |  | 65 | 91 (36, 166) | 0.6 (0.2, 1.1) |  | 252 (97, 545) | 0.6 (0.2, 1.3) |  | 43 (16, 88) | 0.4 (0.1, 0.7) |
|  |  |  | 75+ | 150 (59, 296) | 0.3 (0.1, 0.6) |  | 182 (72, 362) | 0.2 (0.1, 0.4) |  | 71 (27, 146) | 0.2 (0.1, 0.4) |
|  |  | Females | 25 | 1 (1, 2) | 0.5 (0.2, 0.8) |  | 3 (1, 6) | 0.4 (0.2, 0.7) |  | 0 (0, 1) | 0.3 (0.1, 0.4) |
|  |  |  | 35 | 4 (2, 8) | 0.5 (0.2, 1.0) |  | 15 (6, 26) | 0.5 (0.2, 1.0) |  | 1 (0, 2) | 0.2 (0.1, 0.4) |
|  |  |  | 45 | 10 (4, 18) | 0.4 (0.2, 0.7) |  | 53 (21, 109) | 0.6 (0.2, 1.2) |  | 4 (2, 8) | 0.3 (0.1, 0.6) |
|  |  |  | 55 | 24 (10, 46) | 0.5 (0.2, 0.9) |  | 69 (31, 128) | 0.4 (0.2, 0.7) |  | 8 (3, 16) | 0.2 (0.1, 0.4) |
|  |  |  | 65 | 35 (14, 64) | 0.3 (0.1, 0.6) |  | 93 (37, 184) | 0.3 (0.1, 0.6) |  | 13 (5, 26) | 0.3 (0.1, 0.5) |
|  |  |  | 75+ | 109 (44, 213) | 0.2 (0.1, 0.3) |  | 198 (80, 386) | 0.1 (0.1, 0.3) |  | 21 (8, 46) | 0.1 (0, 0.2) |
|  |  |  |  |  |  |  |  |  |  |  |  |
| **Read meats,** | Diabetes | Males | 25 | 0 (0, 1) | 0.5 (0.1, 1.1) |  | 1 (0, 3) | 0.5 (0.1, 1.2) |  | 0 (0, 0) | 0.4 (0, 1) |
| **unprocessed** |  |  | 35 | 2 (0, 4) | 0.7 (0.1, 1.6) |  | 3 (0, 8) | 0.5 (0.1, 1.1) |  | 0 (0, 1) | 0.4 (0.1, 0.9) |
|  |  |  | 45 | 3 (1, 8) | 0.5 (0.1, 1.2) |  | 9 (2, 22) | 0.4 (0.1, 1) |  | 1 (0, 2) | 0.3 (0, 0.7) |
|  |  |  | 55 | 5 (1, 11) | 0.4 (0.1, 0.8) |  | 15 (2, 36) | 0.3 (0, 0.8) |  | 2 (0, 5) | 0.2 (0, 0.5) |
|  |  |  | 65 | 6 (1, 16) | 0.3 (0, 0.7) |  | 13 (2, 31) | 0.2 (0, 0.6) |  | 3 (0, 7) | 0.2 (0, 0.5) |
|  |  |  | 75+ | 6 (1, 13) | 0.1 (0, 0.3) |  | 12 (2, 25) | 0.1 (0, 0.3) |  | 2 (1, 6) | 0.1 (0, 0.2) |
|  |  | Females | 25 | 0 (0, 0) | 0.3 (0.1, 0.8) |  | 1 (0, 1) | 0.4 (0.1, 0.9) |  | 0 (0, 0) | 0.2 (0, 0.6) |
|  |  |  | 35 | 1 (0, 1) | 0.4 (0.1, 1.1) |  | 1 (0, 3) | 0.3 (0, 0.8) |  | 0 (0, 0) | 0.2 (0, 0.5) |
|  |  |  | 45 | 1 (0, 4) | 0.3 (0.1, 0.9) |  | 4 (1, 10) | 0.3 (0.1, 0.8) |  | 0 (0, 1) | 0.2 (0, 0.5) |
|  |  |  | 55 | 2 (0, 6) | 0.3 (0, 0.6) |  | 6 (1, 14) | 0.2 (0, 0.5) |  | 1 (0, 2) | 0.1 (0, 0.3) |
|  |  |  | 65 | 3 (1, 8) | 0.2 (0, 0.5) |  | 7 (1, 17) | 0.2 (0, 0.4) |  | 1 (0, 3) | 0.2 (0, 0.4) |
|  |  |  | 75+ | 6 (1, 15) | 0.1 (0, 0.2) |  | 11 (2, 24) | 0.1 (0, 0.2) |  | 1 (0, 3) | 0.1 (0, 0.2) |
|  |  |  |  |  |  |  |  |  |  |  |  |
| **Sugar-** | CHD | Males | 25 | 11 (5, 21) | 6.8 (2.7, 12.4) |  | 43 (17, 88) | 8.3 (3.4, 17.1) |  | 2 (1, 4) | 2.7 (1.2, 5.0) |
| **sweetened** |  |  | 35 | 53 (22, 103) | 7.3 (2.9, 14.1) |  | 158 (67, 341) | 6.0 (2.5, 13) |  | 11 (5, 25) | 2.7 (1.1, 5.9) |
| **beverages** |  |  | 45 | 112 (48, 205) | 3.5 (1.5, 6.4) |  | 330 (126, 637) | 3.0 (1.2, 5.8) |  | 49 (18, 163) | 2.6 (1.0, 8.8) |
|  |  |  | 55 | 158 (66, 301) | 2.6 (1.1, 5.0) |  | 386 (161, 686) | 1.7 (0.7, 3.1) |  | 53 (24, 103) | 1.0 (0.4, 1.9) |
|  |  |  | 65 | 104 (42, 191) | 1.2 (0.5, 2.1) |  | 244 (103, 460) | 1.0 (0.4, 1.9) |  | 30 (14, 56) | 0.4 (0.2, 0.8) |
|  |  |  | 75+ | 167 (80, 276) | 0.6 (0.3, 1.0) |  | 243 (112, 424) | 0.4 (0.2, 0.7) |  | 62 (28, 113) | 0.3 (0.1, 0.5) |
|  |  | Females | 25 | 4 (2, 7) | 6.6 (2.8, 12.4) |  | 12 (4, 31) | 6.9 (2.6, 18) |  | 1 (0, 1) | 1.9 (0.8, 3.8) |
|  |  |  | 35 | 31 (10, 112) | 10.5 (3.5, 38.3) |  | 65 (24, 189) | 7.1 (2.7, 20.9) |  | 3 (1, 5) | 2.1 (0.9, 3.9) |
|  |  |  | 45 | 53 (21, 106) | 4.5 (1.7, 9.0) |  | 106 (46, 213) | 2.8 (1.2, 5.7) |  | 5 (2, 9) | 1.0 (0.4, 1.7) |
|  |  |  | 55 | 37 (17, 73) | 1.5 (0.7, 3.0) |  | 190 (76, 414) | 2.2 (0.9, 4.7) |  | 11 (4, 21) | 0.7 (0.3, 1.3) |
|  |  |  | 65 | 61 (27, 117) | 1.3 (0.6, 2.4) |  | 113 (47, 207) | 0.8 (0.3, 1.5) |  | 8 (3, 15) | 0.4 (0.1, 0.7) |
|  |  |  | 75+ | 163 (73, 305) | 0.5 (0.2, 0.9) |  | 284 (119, 529) | 0.4 (0.2, 0.7) |  | 23 (7, 48) | 0.2 (0.1, 0.4) |
|  | Hypertensive HD | Males | 25 | 0 (0, 1) | 0.5 (0, 1.8) |  | 1 (0, 4) | 0.6 (0, 2.0) |  | 0 (0, 0) | 0.2 (0, 0.7) |
|  |  |  | 35 | 1 (0, 3) | 0.5 (0, 1.5) |  | 3 (0, 9) | 0.4 (0, 1.3) |  | 0 (0, 1) | 0.2 (0, 0.5) |
|  |  |  | 45 | 2 (0, 5) | 0.3 (0, 0.9) |  | 4 (0, 12) | 0.2 (0, 0.7) |  | 0 (0, 2) | 0.1 (0, 0.5) |
|  |  |  | 55 | 2 (0, 4) | 0.3 (0, 0.6) |  | 5 (1, 12) | 0.2 (0, 0.4) |  | 1 (0, 2) | 0.1 (0, 0.3) |
|  |  |  | 65 | 1 (0, 2) | 0.1 (0.1, 0.3) |  | 2 (1, 4) | 0.1 (0, 0.2) |  | 0 (0, 1) | 0.1 (0, 0.1) |
|  |  |  | 75+ | 2 (0, 4) | 0.1 (0, 0.2) |  | 2 (1, 5) | 0.1 (0, 0.1) |  | 1 (0, 1) | 0 (0, 0.1) |
|  |  | Females | 25 | 0 (0, 0) | 0.4 (0, 1.5) |  | 0 (0, 1) | 0.4 (0, 1.4) |  | 0 (0, 0) | 0.1 (0, 0.5) |
|  |  |  | 35 | 0 (0, 1) | 0.5 (0, 1.6) |  | 1 (0, 3) | 0.3 (0, 1.0) |  | 0 (0, 0) | 0.1 (0, 0.4) |
|  |  |  | 45 | 1 (0, 2) | 0.3 (0, 0.8) |  | 2 (0, 6) | 0.2 (0, 0.6) |  | 0 (0, 0) | 0.1 (0, 0.3) |
|  |  |  | 55 | 0 (0, 1) | 0.1 (0, 0.4) |  | 2 (0, 5) | 0.2 (0, 0.4) |  | 0 (0, 0) | 0.1 (0, 0.1) |
|  |  |  | 65 | 1 (0, 1) | 0.1 (0, 0.3) |  | 1 (0, 2) | 0.1 (0, 0.2) |  | 0 (0, 0) | 0 (0, 0.1) |
|  |  |  | 75+ | 2 (1, 5) | 0.1 (0, 0.2) |  | 4 (1, 9) | 0.1 (0, 0.1) |  | 0 (0, 1) | 0 (0, 0.1) |
|  | Ischemic | Males | 25 | 0 (0, 0) | 0.5 (0.2, 0.9) |  | 0 (0, 0) | 0.5 (0.2, 1) |  | 0 (0, 0) | 0.2 (0.1, 0.4) |
|  | stroke |  | 35 | 0 (0, 0) | 0.4 (0.2, 0.8) |  | 0 (0, 0) | 0.3 (0.1, 0.6) |  | 0 (0, 0) | 0.1 (0.1, 0.3) |
|  |  |  | 45 | 0 (0, 0) | 0.2 (0.1, 0.4) |  | 0 (0, 1) | 0.2 (0.1, 0.3) |  | 0 (0, 0) | 0.1 (0, 0.2) |
|  |  |  | 55 | 0 (0, 0) | 0.2 (0.1, 0.3) |  | 1 (0, 1) | 0.1 (0, 0.2) |  | 0 (0, 0) | 0.1 (0, 0.2) |
|  |  |  | 65 | 0 (0, 0) | 0.1 (0, 0.1) |  | 0 (0, 1) | 0.1 (0, 0.1) |  | 0 (0, 0) | 0 (0, 0.1) |
|  |  |  | 75+ | 0 (0, 1) | 0 (0, 0) |  | 0 (0, 1) | 0 (0, 0) |  | 0 (0, 0) | 0 (0, 0) |
|  |  | Females | 25 | 0 (0, 0) | 0.4 (0.2, 0.8) |  | 0 (0, 0) | 0.3 (0.1, 0.6) |  | 0 (0, 0) | 0.1 (0, 0.2) |
|  |  |  | 35 | 0 (0, 0) | 0.4 (0.2, 0.7) |  | 0 (0, 0) | 0.3 (0.1, 0.5) |  | 0 (0, 0) | 0.1 (0, 0.2) |
|  |  |  | 45 | 0 (0, 0) | 0.2 (0.1, 0.4) |  | 0 (0, 0) | 0.2 (0.1, 0.3) |  | 0 (0, 0) | 0.1 (0, 0.1) |
|  |  |  | 55 | 0 (0, 0) | 0.1 (0, 0.2) |  | 0 (0, 1) | 0.1 (0, 0.2) |  | 0 (0, 0) | 0 (0, 0.1) |
|  |  |  | 65 | 0 (0, 0) | 0.1 (0, 0.1) |  | 0 (0, 0) | 0 (0, 0.1) |  | 0 (0, 0) | 0 (0, 0) |
|  |  |  | 75+ | 0 (0, 1) | 0 (0, 0) |  | 1 (0, 1) | 0 (0, 0) |  | 0 (0, 0) | 0 (0, 0) |
|  | Hemorrhagic | Males | 25 | 0 (0, 1) | 0.7 (0.3, 1.4) |  | 1 (0, 2) | 0.8 (0.3, 1.5) |  | 0 (0, 0) | 0.3 (0.1, 0.6) |
|  | stroke |  | 35 | 1 (0, 2) | 0.6 (0.3, 1.3) |  | 2 (1, 4) | 0.5 (0.2, 1.0) |  | 0 (0, 0) | 0.2 (0.1, 0.4) |
|  |  |  | 45 | 1 (1, 3) | 0.3 (0.1, 0.7) |  | 3 (1, 5) | 0.3 (0.1, 0.5) |  | 0 (0, 1) | 0.2 (0.1, 0.4) |
|  |  |  | 55 | 1 (0, 2) | 0.2 (0.1, 0.5) |  | 3 (1, 5) | 0.2 (0.1, 0.3) |  | 0 (0, 1) | 0.1 (0, 0.2) |
|  |  |  | 65 | 0 (0, 1) | 0.1 (0, 0.2) |  | 1 (0, 3) | 0.1 (0, 0.2) |  | 0 (0, 1) | 0 (0, 0.1) |
|  |  |  | 75+ | 0 (0, 1) | 0 (0, 0.1) |  | 1 (0, 2) | 0 (0, 0) |  | 0 (0, 0) | 0 (0, 0) |
|  |  | Females | 25 | 0 (0, 0) | 0.6 (0.2, 1.2) |  | 1 (0, 1) | 0.5 (0.2, 1.0) |  | 0 (0, 0) | 0.2 (0.1, 0.4) |
|  |  |  | 35 | 1 (0, 1) | 0.6 (0.2, 1.2) |  | 1 (1, 3) | 0.4 (0.2, 0.8) |  | 0 (0, 0) | 0.2 (0.1, 0.3) |
|  |  |  | 45 | 1 (0, 2) | 0.3 (0.1, 0.6) |  | 3 (1, 5) | 0.2 (0.1, 0.5) |  | 0 (0, 0) | 0.1 (0, 0.2) |
|  |  |  | 55 | 0 (0, 1) | 0.1 (0.1, 0.3) |  | 2 (1, 4) | 0.1 (0.1, 0.3) |  | 0 (0, 0) | 0 (0, 0.1) |
|  |  |  | 65 | 0 (0, 1) | 0.1 (0, 0.2) |  | 1 (0, 2) | 0.1 (0, 0.1) |  | 0 (0, 0) | 0 (0, 0.1) |
|  |  |  | 75+ | 0 (0, 1) | 0 (0, 0) |  | 1 (0, 2) | 0 (0, 0) |  | 0 (0, 0) | 0 (0, 0) |
|  | Other stroke | Males | 25 | 0 (0, 0) | 0.7 (0.3, 1.4) |  | 0 (0, 0) | 0.8 (0.3, 1.5) |  | 0 (0, 0) | 0.3 (0.1, 0.6) |
|  |  |  | 35 | 0 (0, 0) | 0.6 (0.3, 1.2) |  | 1 (0, 2) | 0.5 (0.2, 0.9) |  | 0 (0, 0) | 0.2 (0.1, 0.4) |
|  |  |  | 45 | 1 (0, 2) | 0.3 (0.1, 0.6) |  | 2 (1, 3) | 0.2 (0.1, 0.5) |  | 0 (0, 0) | 0.2 (0.1, 0.3) |
|  |  |  | 55 | 1 (0, 3) | 0.2 (0.1, 0.4) |  | 3 (1, 6) | 0.2 (0.1, 0.3) |  | 0 (0, 1) | 0.1 (0, 0.2) |
|  |  |  | 65 | 1 (0, 2) | 0.1 (0, 0.2) |  | 2 (1, 5) | 0.1 (0, 0.1) |  | 0 (0, 1) | 0 (0, 0.1) |
|  |  |  | 75+ | 1 (0, 3) | 0 (0, 0.1) |  | 2 (1, 4) | 0 (0, 0) |  | 0 (0, 1) | 0 (0, 0) |
|  |  | Females | 25 | 0 (0, 0) | 0.6 (0.2, 1.2) |  | 0 (0, 0) | 0.5 (0.2, 1.0) |  | 0 (0, 0) | 0.2 (0.1, 0.3) |
|  |  |  | 35 | 0 (0, 1) | 0.6 (0.2, 1.2) |  | 0 (0, 1) | 0.4 (0.2, 0.8) |  | 0 (0, 0) | 0.2 (0.1, 0.3) |
|  |  |  | 45 | 1 (0, 1) | 0.3 (0.1, 0.6) |  | 1 (0, 2) | 0.2 (0.1, 0.4) |  | 0 (0, 0) | 0.1 (0, 0.2) |
|  |  |  | 55 | 1 (0, 1) | 0.1 (0, 0.3) |  | 2 (1, 4) | 0.1 (0, 0.3) |  | 0 (0, 0) | 0 (0, 0.1) |
|  |  |  | 65 | 1 (0, 2) | 0.1 (0, 0.2) |  | 1 (1, 3) | 0.1 (0, 0.1) |  | 0 (0, 0) | 0 (0, 0.1) |
|  |  |  | 75+ | 2 (1, 4) | 0 (0, 0) |  | 3 (1, 8) | 0 (0, 0) |  | 0 (0, 1) | 0 (0, 0) |
|  | Stroke, total | Males | 25 | 0 (0, 1) | 0.7 (0.4, 1.3) |  | 2 (1, 3) | 0.8 (0.4, 1.4) |  | 0 (0, 0) | 0.3 (0.2, 0.5) |
|  |  |  | 35 | 1 (1, 2) | 0.6 (0.3, 1.1) |  | 3 (2, 5) | 0.5 (0.3, 0.8) |  | 0 (0, 0) | 0.2 (0.1, 0.4) |
|  |  |  | 45 | 2 (1, 4) | 0.3 (0.2, 0.5) |  | 5 (3, 8) | 0.3 (0.1, 0.4) |  | 1 (0, 1) | 0.2 (0.1, 0.3) |
|  |  |  | 55 | 3 (1, 5) | 0.2 (0.1, 0.4) |  | 6 (3, 10) | 0.2 (0.1, 0.2) |  | 1 (1, 2) | 0.1 (0.1, 0.2) |
|  |  |  | 65 | 2 (1, 3) | 0.1 (0, 0.1) |  | 4 (2, 7) | 0.1 (0, 0.1) |  | 1 (0, 1) | 0 (0, 0.1) |
|  |  |  | 75+ | 2 (1, 4) | 0 (0, 0) |  | 3 (1, 5) | 0 (0, 0) |  | 1 (0, 1) | 0 (0, 0) |
|  |  | Females | 25 | 0 (0, 0) | 0.6 (0.3, 1.1) |  | 1 (0, 1) | 0.5 (0.3, 0.8) |  | 0 (0, 0) | 0.2 (0.1, 0.3) |
|  |  |  | 35 | 1 (0, 2) | 0.6 (0.3, 1.0) |  | 2 (1, 3) | 0.4 (0.2, 0.7) |  | 0 (0, 0) | 0.2 (0.1, 0.3) |
|  |  |  | 45 | 2 (1, 3) | 0.3 (0.2, 0.5) |  | 4 (2, 6) | 0.2 (0.1, 0.4) |  | 0 (0, 0) | 0.1 (0.1, 0.2) |
|  |  |  | 55 | 1 (1, 2) | 0.1 (0.1, 0.2) |  | 4 (2, 7) | 0.1 (0.1, 0.2) |  | 0 (0, 1) | 0 (0, 0.1) |
|  |  |  | 65 | 2 (1, 3) | 0.1 (0, 0.1) |  | 3 (2, 4) | 0.1 (0, 0.1) |  | 0 (0, 0) | 0 (0, 0) |
|  |  |  | 75+ | 3 (1, 5) | 0 (0, 0) |  | 5 (3, 10) | 0 (0, 0) |  | 0 (0, 1) | 0 (0, 0) |
|  | Diabetes | Males | 25 | 6 (2, 13) | 7.4 (2.5, 16.7) |  | 22 (8, 58) | 9.6 (3.3, 25) |  | 1 (0, 2) | 3.1 (1.1, 6.5) |
|  |  |  | 35 | 20 (7, 51) | 8.3 (3.0, 20.9) |  | 50 (16, 138) | 6.8 (2.1, 18.8) |  | 3 (1, 8) | 3.1 (1.0, 8.8) |
|  |  |  | 45 | 28 (10, 56) | 3.9 (1.4, 8.0) |  | 77 (28, 165) | 3.4 (1.2, 7.3) |  | 10 (3, 63) | 3.1 (0.8, 18.7) |
|  |  |  | 55 | 39 (14, 97) | 2.9 (1.0, 7.4) |  | 88 (35, 179) | 1.9 (0.8, 3.9) |  | 11 (4, 22) | 1.1 (0.4, 2.2) |
|  |  |  | 65 | 28 (11, 57) | 1.3 (0.5, 2.7) |  | 58 (24, 125) | 1.1 (0.5, 2.4) |  | 7 (2, 12) | 0.5 (0.2, 0.9) |
|  |  |  | 75+ | 30 (13, 57) | 0.7 (0.3, 1.3) |  | 38 (17, 69) | 0.5 (0.2, 0.9) |  | 8 (4, 16) | 0.3 (0.2, 0.7) |
|  |  | Females | 25 | 4 (1, 10) | 7.4 (2.6, 17.1) |  | 14 (4, 52) | 8.1 (2.3, 30.3) |  | 0 (0, 1) | 2.3 (0.7, 5.4) |
|  |  |  | 35 | 17 (4, 91) | 12.9 (3.2, 67.1) |  | 39 (10, 205) | 8.7 (2.1, 45.7) |  | 1 (0, 3) | 2.3 (0.8, 5.7) |
|  |  |  | 45 | 21 (6, 51) | 4.9 (1.5, 12.0) |  | 44 (15, 109) | 3.2 (1.1, 7.9) |  | 2 (1, 4) | 1.0 (0.4, 2.2) |
|  |  |  | 55 | 16 (5, 32) | 1.7 (0.6, 3.6) |  | 71 (21, 196) | 2.4 (0.7, 6.6) |  | 4 (1, 9) | 0.7 (0.3, 1.7) |
|  |  |  | 65 | 23 (7, 51) | 1.4 (0.4, 3.0) |  | 37 (14, 79) | 0.9 (0.3, 2.0) |  | 3 (1, 5) | 0.4 (0.1, 0.8) |
|  |  |  | 75+ | 33 (14, 64) | 0.5 (0.2, 1.0) |  | 46 (20, 86) | 0.4 (0.2, 0.8) |  | 3 (1, 7) | 0.2 (0.1, 0.5) |
|  | CMD, total | Males | 25 | 19 (10, 30) | 3.6 (1.9, 5.6) |  | 71 (36, 121) | 4.4 (2.3, 7.5) |  | 3 (2, 5) | 1.3 (0.7, 2.1) |
|  |  |  | 35 | 79 (40, 131) | 4.8 (2.4, 8.0) |  | 219 (119, 413) | 3.8 (2.1, 7.2) |  | 15 (8, 28) | 1.6 (0.8, 3.0) |
|  |  |  | 45 | 146 (75, 241) | 2.5 (1.3, 4.2) |  | 422 (206, 748) | 2.2 (1.1, 3.9) |  | 64 (28, 239) | 2.0 (0.9, 7.3) |
|  |  |  | 55 | 206 (108, 351) | 2.0 (1.1, 3.4) |  | 490 (260, 799) | 1.3 (0.7, 2.1) |  | 66 (37, 116) | 0.7 (0.4, 1.3) |
|  |  |  | 65 | 134 (73, 228) | 0.9 (0.5, 1.5) |  | 310 (165, 542) | 0.7 (0.4, 1.3) |  | 38 (20, 64) | 0.3 (0.2, 0.5) |
|  |  |  | 75+ | 203 (109, 325) | 0.4 (0.2, 0.7) |  | 288 (152, 477) | 0.3 (0.2, 0.5) |  | 72 (37, 124) | 0.2 (0.1, 0.3) |
|  |  | Females | 25 | 9 (5, 15) | 3.4 (1.9, 6.1) |  | 28 (14, 71) | 3.5 (1.7, 8.8) |  | 1 (1, 2) | 0.8 (0.4, 1.3) |
|  |  |  | 35 | 55 (23, 156) | 6.8 (2.8, 19.3) |  | 116 (53, 315) | 4.3 (1.9, 11.6) |  | 4 (2, 7) | 0.8 (0.4, 1.4) |
|  |  |  | 45 | 78 (40, 138) | 3.0 (1.5, 5.2) |  | 161 (84, 280) | 1.8 (0.9, 3.1) |  | 7 (4, 11) | 0.5 (0.3, 0.8) |
|  |  |  | 55 | 56 (30, 93) | 1.1 (0.6, 1.8) |  | 278 (139, 563) | 1.5 (0.8, 3.0) |  | 16 (8, 28) | 0.4 (0.2, 0.8) |
|  |  |  | 65 | 88 (46, 149) | 0.9 (0.5, 1.5) |  | 156 (84, 257) | 0.6 (0.3, 0.9) |  | 11 (6, 19) | 0.2 (0.1, 0.4) |
|  |  |  | 75+ | 204 (110, 350) | 0.3 (0.2, 0.5) |  | 342 (171, 596) | 0.2 (0.1, 0.4) |  | 27 (12, 58) | 0.1 (0, 0.2) |
|  |  |  |  |  |  |  |  |  |  |  |  |

^a^ Estimated using nationally representative data from the US adult population in 2012 based on a comparative risk assessment framework (fruits, vegetables, nuts/seeds, whole grains, processed meat, unprocessed red meat, and sugar-sweetened beverages). Estimates based on a low SES gradient (18.2% differential effect comparing those with < high school versus college education).

^b^ CVD corresponds to the sum of CHD, hypertensive heart disease and stroke; and CMD to the sum of CVD and diabetes. Values may not precisely sum due to rounding.

**Table S4. Mortality rates (2012)*^a^* among US adults and disparities by education level**

| **Outcome** | **Education**  **level** | **Mortality**  **(deaths per million)** | **% Disparity**  **(<HS vs COL)*^b^*** |
| --- | --- | --- | --- |
|  |  |  |  |
| **CHD** | <HS | 3143 | 112 |
|  | HS | 1839 |  |
|  | COL | 882 |  |
|  |  |  |  |
| **Hypertensive HD** | <HS | 280 | 109 |
|  | HS | 176 |  |
|  | COL | 82 |  |
|  |  |  |  |
| **Stroke** | <HS | 1115 | 111 |
|  | HS | 627 |  |
|  | COL | 317 |  |
|  |  |  |  |
| **Diabetes** | <HS | 636 | 129 |
|  | HS | 335 |  |
|  | COL | 136 |  |
|  |  |  |  |
| **CMD** | <HS | 5954 | 112 |
|  | HS | 3480 |  |
|  | COL | 1679 |  |

^a^ Data on US deaths by education were derived from the National Center for Health Statistics, including deaths due to CHD (ICD10: I20-I25), stroke (I60-I62, I63, I64, I65-I67, I69.0-I69.2, I69.3, I69.4, I69.8, G45), diabetes mellitus (E10-E14 (except E10.2, E11.2, E12.2, E13.2), and hypertensive heart disease (HHD) (I11). ^b^ Calculated as the percent difference according to the formula % = |ΔV|ΣV/2×100

**Figure S1**. Distribution of educational level and family income among US adults ^a^

^a^ Based on nationally-representative data combining the 2009-2010 and 2011-2012 cycles of the National Health and Nutrition Examination Survey (NHANES) for the adult US population (age 25+ years; N=8,516), accounting for complex survey design and sampling weights as appropriate, and adjusted by family size.
